# Systematic Analysis of Gibberellin Pathway Components in *Medicago truncatula* Reveals the Potential Application of Gibberellin in Biomass Improvement

**DOI:** 10.3390/ijms21197180

**Published:** 2020-09-29

**Authors:** Hongfeng Wang, Hongjiao Jiang, Yiteng Xu, Yan Wang, Lin Zhu, Xiaolin Yu, Fanjiang Kong, Chuanen Zhou, Lu Han

**Affiliations:** 1 School of Life Science, Guangzhou University, Guangzhou 510006, China; kongfj@iga.ac.cn; 2 The Key Laboratory of Plant Development and Environmental Adaptation Biology, Ministry of Education, School of Life Science, Shandong University, Qingdao 266101, China; 201611650@mail.sdu.edu.cn (H.J.); 201411612@mail.sdu.edu.cn (Y.X.); 201812415@mail.sdu.edu.cn (Y.W.); 201832474@mail.sdu.edu.cn (L.Z.); yuxiaolin@sdu.edu.cn (X.Y.); czhou@sdu.edu.cn (C.Z.)

**Keywords:** *Medicago truncatula*, gibberellins, expression analysis, forage improvement

## Abstract

Gibberellins (GAs), a class of phytohormones, act as an essential natural regulator of plant growth and development. Many studies have shown that GA is related to rhizobial infection and nodule organogenesis in legume species. However, thus far, GA metabolism and signaling components are largely unknown in the model legume *Medicago truncatula*. In this study, a genome-wide analysis of GA metabolism and signaling genes was carried out. In total 29 components, including 8 *MtGA20ox* genes, 2 *MtGA3ox* genes, 13 *MtGA2ox* genes, 3 *MtGID1* genes, and 3 *MtDELLA* genes were identified in *M. truncatula* genome. Expression profiles revealed that most members of *MtGAox*, *MtGID1*, and *MtDELLA* showed tissue-specific expression patterns. In addition, the GA biosynthesis and deactivation genes displayed a feedback regulation on GA treatment, respectively. Yeast two-hybrid assays showed that all the three MtGID1s interacted with MtDELLA1 and MtDELLA2, suggesting that the MtGID1s are functional GA receptors. More importantly, *M. truncatula* exhibited increased plant height and biomass by ectopic expression of the *MtGA20ox1*, suggesting that enhanced GA response has the potential for forage improvement.

## 1. Introduction

Gibberellins (GAs), a class of well-known phytohormones, play important roles in regulating growth and development throughout the life cycle, such as seed germination, stem/internode elongation, leaf expansion, flowering, fruit development, and nodule organogenesis in legume [1,2,3,4,5,6,7,8]. Accumulating evidence has elucidated the molecular mechanism of GA biosynthesis, metabolism, and signaling in plants [9,10,11,12]. In higher plants, GA synthesis can be divided into three steps (Figure S1): (i) biosynthesis of *ent*-kaurene from *trans*-geranyl-geranyl diphosphate (*trans*-GGDP) by *ent*-copalyl diphosphate synthase (CPS) and *ent*-kaurene synthase (KS) in plastids; (ii) conversion of *ent*-kaurene to *ent*-kaurenic acid by *ent*-kaurene oxidase (KO), and (iii) conversion of *ent*-kaurenic acid to bioactive GAs by *ent*-kaurenoic acid oxidase (KAO), GA 20-oxidase (GA20ox), and GA 3-oxidase (GA3ox). Degradation of GA is mainly catalyzed by GA 2-oxidase (GA2ox). GA20ox, GA3ox and GA2ox proteins all belonged to the 2-oxoglutarate-dependent dioxygenase (2-ODDs) family, which containing two domains, DIOX_N and 2OG-FeII_Oxy. The 2OG-FeII_Oxy domain contains the consensus sequence NXYPXCXXP and three histidine residues, which are respectively involved in binding the common co-substrate 2-oxoglutarate and Fe^2+^. The DIOX_N domain contains the LPWKET sequence related to the binding of the GA substrate. Reduction of endogenous bioactive GA content by mutation and down-regulation of *GA20ox* can generate dwarf phenotypes in diverse species and contributed to the Green Revolution. In rice, *SD1* encodes a GA20-oxidase and its mutants show dwarfism and increase in yield [13]. In *Arabidopsis*, a total of five *GA20ox*, *AtGA20ox1* to *AtGA20ox5*, are identified and loss-of-function in *AtGA20ox1*, *-2*, and *-3* results in mutants with severe dwarfism, dark green leaves, and delayed flowering [14]. Furthermore, *GA3ox* also participates in the synthesis of bioactive GAs and the *ga3ox1 ga3ox2* double mutants display defects in shoot and root development [15]. Similar to the *ga20ox* and *ga3ox* mutants, overexpression of *BnGA2ox6* and *JcGA2ox6* in *Arabidopsis* results in GA-deficiency phenotypes, including late flowering, smaller leaves, and shorter siliques [16,17]. However, ectopic overexpression of the *AtGA20ox1* gene in hybrid aspen results in trees with longer xylem fibers, larger leaves, and increased biomass [18]. Several biochemical and molecular evidence had shown that the transcript levels of *GA20ox* and *GA3ox* are regulated by the negative feedback of bioactive GAs, while *GA2ox* is regulated by positive feedback [1,19]. Besides, GA biosynthesis and metabolism are regulated by diverse environmental signals, such as light and cold [20,21].

Once GA is synthesized and present, the GA signal transduction is mediated by GIBBERELLIN INSENSITIVE DWARF 1 (GID1) receptor and DELLA protein. Briefly, GA binding to the soluble GID1 receptor triggers its interaction with DELLA proteins; subsequently DELLA is subject to polyubiquitination by E3 ubiquitin-ligase SCF^SLY1/GID2^ and degradation in the 26S proteasome [22,23,24,25,26,27,28,29]. The rice GID1 is the first and only one identified GA receptor and loss-of-function *gid1* mutations result in a GA-insensitive dwarf phenotype [22,30,31]. Plants overproducing *OsGID1* show a GA-hypersensitive phenotype with tall stature, light-green leaves, and fewer tillers [22]. The *Arabidopsis* genome contains three GID1 homologs, *AtGID1A*, *AtGID1B*, and *AtGID1C*, which are partially redundant in regulating different developmental processes [32,33,34]. The tomato genome encodes three receptors (SlGID1a, SlGID1b1, and SlGID1b2) and these GA receptors contribute to phenotypic stability under ambient changing environments [35]. DELLA proteins are key regulators of the GA signaling pathway and negatively regulate GA responses [36]. GID1 can directly interact with DELLA domain of DELLA proteins in a GA-dependent manner, and the formation of GA-GID1-DELLA regulatory module promotes the rapid degradation of DELLA repressor [22,32,37,38,39,40]. The *Arabidopsis* genome contains five genes (*GAI*, *RGA*, *RGL1*, *RGL2*, and *RGL3*) encoding DELLA proteins, while rice (*SLR1*) and tomato (*PRO*) have only one [23,41,42,43,44,45]. DELLA proteins are highly conserved in function. Loss-of-function mutants of *RGA*, *SLR1*, and *PRO* show a slender phenotype with stem elongation and leaf expansion [25,43,46,47,48]. While, gain-of-function mutations in *DELLA* genes result in plant dwarfism and reduction in GA response [45,49]. The famous wheat “green revolution” *Rht* genes encode GA-insensitive DELLA proteins that reduce plant height and increase grain number [50,51].

*Medicago truncatula* is a model species for legume studies and its relative alfalfa (*Medicago sativa*) is an important forage crop. Increased biomass yield benefits not only the livestock industry but also sustainable agriculture worldwide. Previous studies have shown that altering GA metabolism or signaling contributes to biomass production [18,52,53,54]. However, knowledge about the molecular features of the GA pathway and response are limited in *M. truncatula*, let alone the relationship between GA and biomass. In this study, GA metabolism and signaling genes were identified and characterized. Moreover, GA application resulted in improved biomass yield in *M. truncatula*. Our results provide a theoretical foundation for GA-dependent forage improvement in the future.

## 2. Results

### 2.1. Genome-Wide Identification of GA20ox, GA3ox, and GA2ox Genes in M. truncatula

To identify the *GA20ox*, *GA3ox*, and *GA2ox* genes in *M. truncatula*, a BLASTP search using the *A. thaliana AtGA20ox*, *AtGA3ox*, and *AtGA2ox* genes against *M. truncatula* database was executed. A total of 23 candidate genes were obtained in the *M. truncatula* genome, including 8 *MtGA20ox*, 2 *MtGA3ox*, and 13 *MtGA2ox* (Table S1). These *MtGAox* genes were named based on their closest *Arabidopsis* orthologs. The gene names, accession numbers, locations, coding sequence (CDS) lengths and the number of the deduced amino acid are summarized in Table S1.

### 2.2. Phylogenetic Analysis and Chromosomal Locations of MtGAox Genes

**Figure 1:**
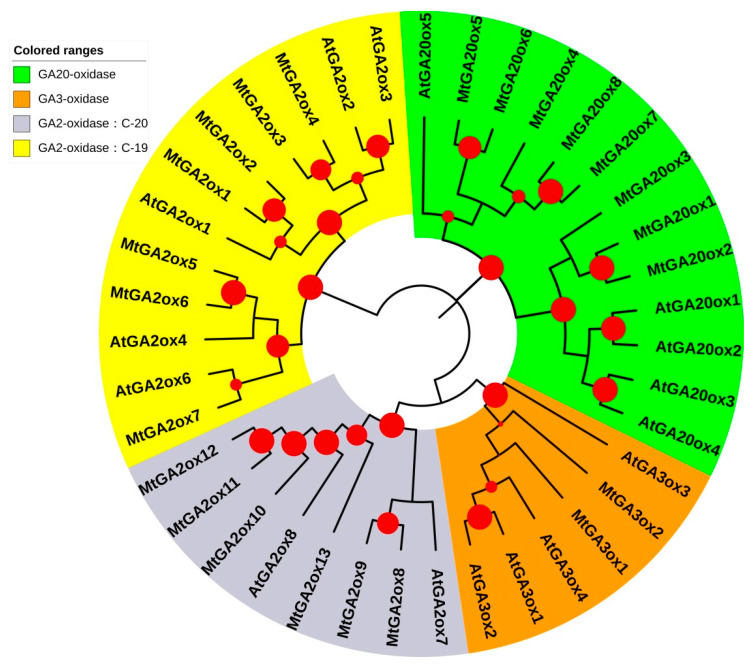
Phylogenetic analysis of GA20oxs, GA3oxs, and GA2oxs in *A. thaliana* (At) and *M.truncatula* (Mt). The proteins are grouped into four distinct groups. The phylogenetic tree was constructed using 39 protein sequences from *A. thaliana* (16) and *M.truncatula* (23) on iTOLv5 (Interactive Tree of Life; https://itol.embl.de/) online website. The red dots of different sizes (Min size: 5 px; Max size: 30 px) represent the bootstrap values, ranging from 0.5 to 1. The branches covered by different color represent the four group proteins.

To establish the evolutionary relationships within the *MtGAox* genes, a phylogenetic tree was constructed using the 39 GAox proteins from *M. truncatula* and *A. thaliana*, including 5 AtGA20ox, 8 MtGA20ox, 4 AtGA3ox, 2 MtGA3ox, 7 AtGA2ox, and 13 MtGA2ox. Based on the phylogenetic analysis, the 39 GAox proteins were divided into four subfamilies (Figure 1): the gibberellin biosynthesis gene family contained the GA20ox and GA3ox genes, and the gibberellin deactivation GA2ox genes could be divided into C19- and C20-GA2ox subgroups.

The 23 *MtGAox* genes were distributed among 8 chromosomes (Figure S2A). The chromosome 02, 01 and 08, 07, 03 and 04 had six, four, three, and two genes, respectively, while the chromosome 05 and 06 each contained only one.

### 2.3. Gene Structures and Conserved Motifs of MtGAox in M. truncatula

To get a better understanding of the structural diversity of the *MtGAox* genes, the structures (exon/intron organization) of *MtGAox* were analyzed (Figure S2B). *MtGA20ox* and *MtGA2ox* genes had the same structure, which all contain three exons. However, all the *MtGA3ox* genes had two exons. Exon-intron organization indicated that *MtGA20ox* and *MtGA2ox* genes were more closely related than *MtGA3ox* genes.

**Figure 2:**
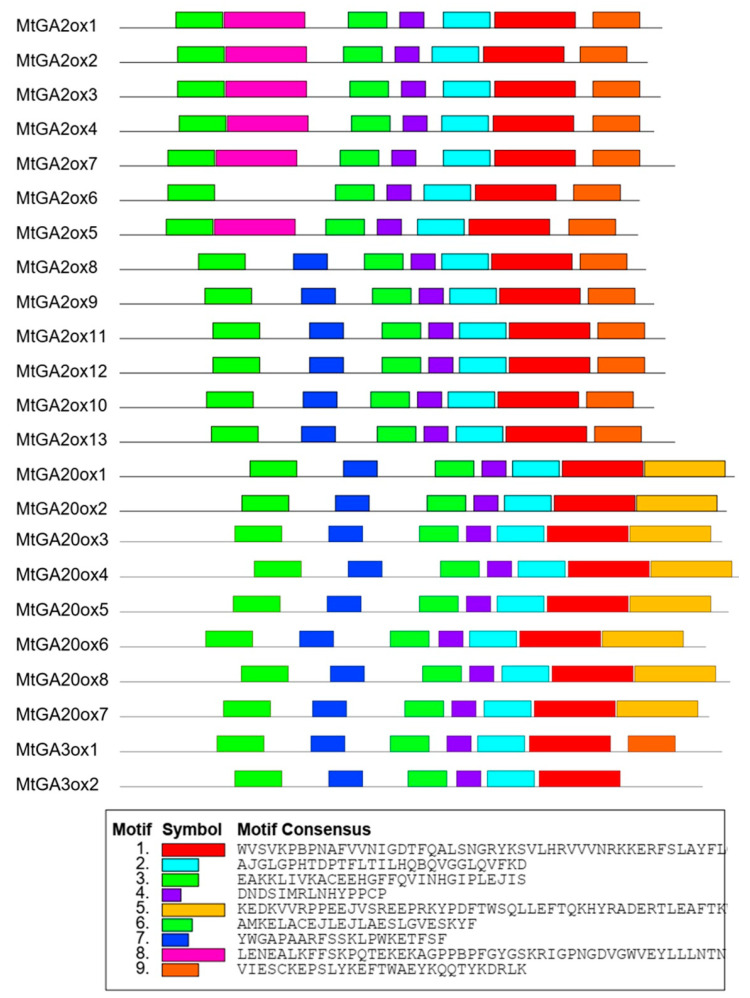
Motifs composition of the MtGA20ox, MtGA3ox, and MtGA2ox proteins. The conserved motifs are predicted by MEME. The different motifs are represented by colored boxes. Motif 3 is representative DIOX_N domain. Motif 1 and motif 2 are representative 2OG-FeII_Oxy domain.

To obtain a better understanding of the protein sequence characteristics of the MtGAox, MEME search was used to analyze the conserved domains and motifs. Based on the composition of motifs, the MtGAox proteins contained nine different motifs (Figure 2; Figure S3). Motif 1, 2, 3, 4, and 6 were identified as the conserved motif, which was present in every MtGAox protein. Motif 1 and 2 are representative 2OG-FeII_Oxy domain and Motif 3 is representative DIOX_N domain, which are identified in all GAox proteins. Motif 5 was only identified in C-terminal of MtGA20ox proteins, indicating that members within a single subfamily usually exhibit similar motif composition. However, motif 8 was located in the C-terminal part of some members of the MtGA2ox protein family, demonstrating that this motif is related to the functions of these MtGA2ox proteins.

### 2.4. Expression Patterns of MtGAox Genes

**Figure 3:**
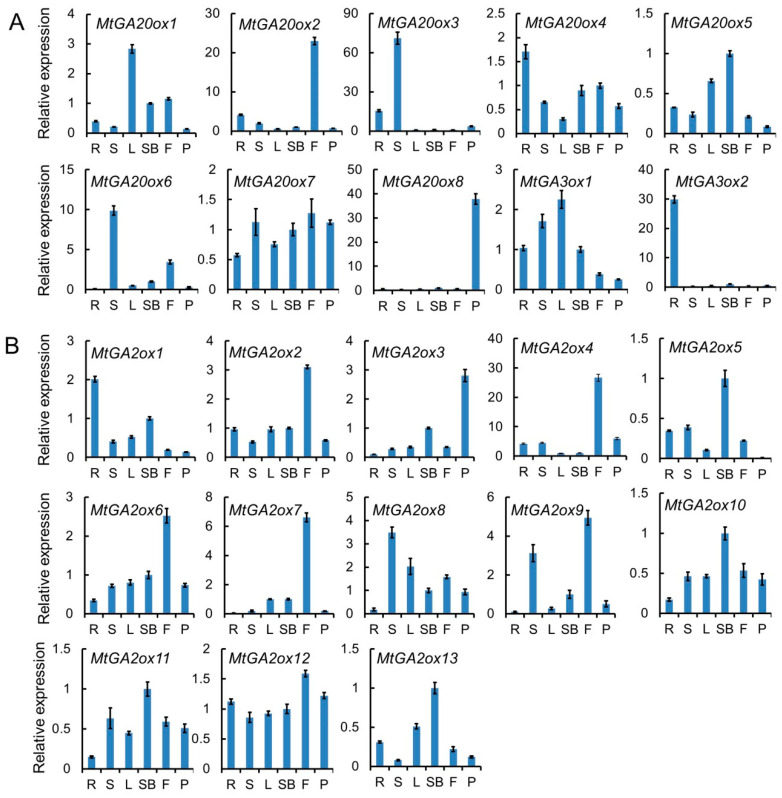
The expression patterns of *MtGA20ox*, *MtGA3ox*, and *MtGA2ox* genes in *M. truncatula*. (**A**) Expression analysis of gibberellin biosynthesis genes *MtGA20ox* and *MtGA3ox* in different tissues. (**B**) Expression analysis of gibberellin deactivation genes *MtGA2ox* in different tissues. R, roots; S, stems; L, leaves; SB, shoot buds; F, flowers; P, pods. The level of expression was normalized to *M. truncatula UBI* gene. Error bars represent SD for three biological replicates.

The tissue-specific expression patterns of a gene family can provide information about its possible function. To understand the functional divergence of the *MtGAox* genes, we analyzed the expression profiles of the *MtGAox* genes in different organs/tissues by quantitative RT-PCR (qRT-PCR) and gene expression atlas (Figure 3; Figures S4 and S5). The results indicated that GA biosynthesis gene *MtGA20ox1*, *MtGA20ox4*, *MtGA20ox5*, *MtGA20ox7*, and *MtGA3ox1* were widely expressed in all tissues examined (Figure 3A). While, *MtGA20ox3* and *MtGA20ox6* had higher expression levels in stems, *MtGA20ox2*, *MtGA20ox8*, and *MtGA3ox2* had higher expression levels in flowers, pods, and roots (Figure 3A), respectively, implying important roles of these genes in certain organs or tissues. Most of the GA deactivation genes, such as *MtGA2ox1*, *MtGA2ox2*, *MtGA2ox6*, *MtGA2ox8*, *MtGA2ox9*, *MtGA2ox10*, *MtGA2ox11*, *MtGA2ox12*, and *MtGA2ox13* were widely expressed in all organs tested (Figure 3B). However, *MtGA2ox4* and *MtGA2ox7* were expressed at higher levels in flowers than those in other tissues (Figure 3B), implying their possible involvement in flower development.

### 2.5. Identification of GA Receptor MtGID1 and MtDELLA Genes in M. truncatula

**Figure 4:**
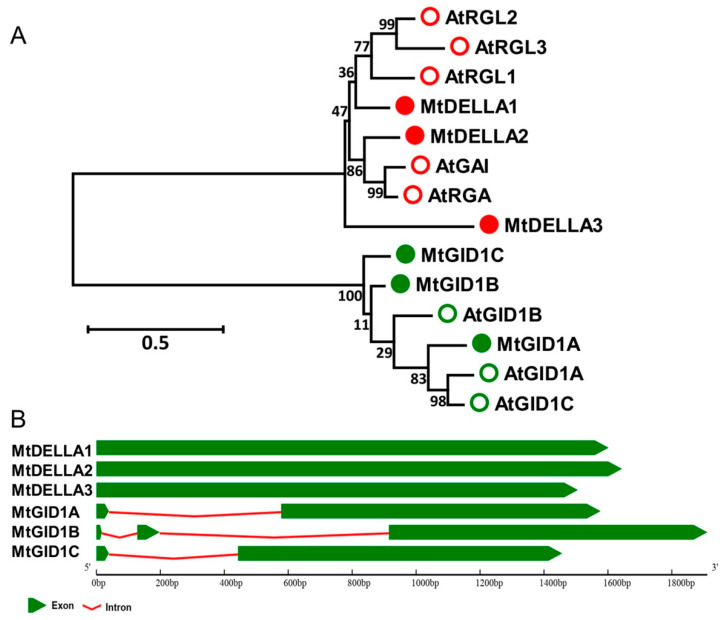
Phylogenetic tree analysis and exon-intron structures of the *DELLA* and *GID1* gene family. (**A**) Phylogenetic analysis of DELLA and GID1 proteins from *A. thaliana* and *M. truncatula*. The tree is generated with MEGA 7.0 software using the Neighbor-Joining (NJ) method. The bootstrap analysis was conducted with 1000 iterations. The values on the phylogenetic tree represent the result of bootstrap analysis conducted with 1000 iterations. The scale bar indicates that the sequence divergence is 0.05 per unit bar, which represent 5% substitutions per nucleotide position. The empty and full circles in red color represent *A. thaliana* and *M. truncatula* DELLA proteins and the empty and full circles in green color represent *A. thaliana* and *M. truncatula* GID1 receptor proteins (**B**) The gene structures of the *MtDELLAs* and *MtGID1s*. Exons are represented by green boxes; introns are shown as red lines.

When GA is present, the GID1 receptors bind to GA and this binding promotes the interaction of GID1 with DELLA proteins, which are degraded in the 26S proteasome later (Figure S6). To identify MtGID1 and MtDELLA proteins in *M. truncatula*, BLASTP searches were performed using 3 AtGID1 and 5 AtDELLA sequences. A total of three GA receptor proteins, MtGID1A, MtGID1B, and MtGID1C, and 3 MtDELLA proteins, MtDELLA1, MtDELLA2, and MtDELLA3, were identified (Table S2; Figure 4A; Figures S7 and S8;). Gene structure analysis showed that the *MtDELLA* genes had the same exon–intron organization with no intron, whereas the intron of *MtGID1* genes ranged from 1 to 2 (Figure 4B).

### 2.6. Expression Patterns of MtGID1 and MtDELLA Genes

**Figure 5:**
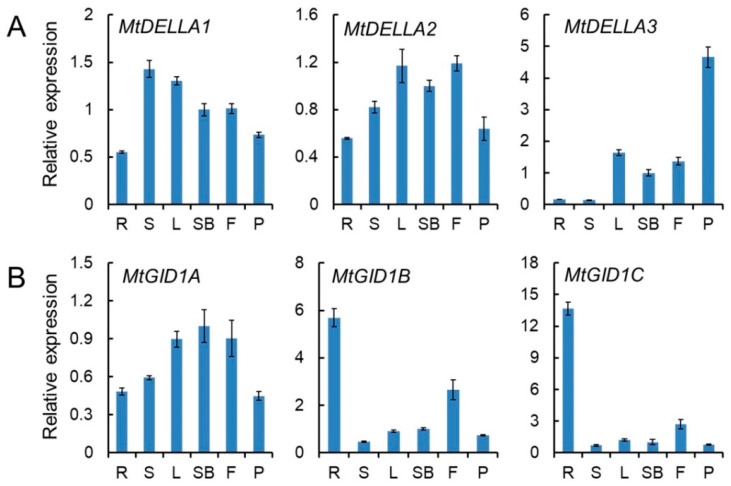
Expression patterns of *MtDELLA* (**A**) and *MtGID1* (**B**) genes in six different tissues. R, roots; S, stems; L, leaves; SB, shoot buds; F, flowers; P, pods. The level of expression was normalized to *M. truncatula UBI* gene. Error bars represent SD for three biological replicates.

To gain better insights into the potential functions of *MtGID1* and *MtDELLA* genes, their expression patterns were analyzed (Figure 5; Figures S9 and S10). The qRT-PCR results indicated that *MtDELLA1* and *MtDELLA2* had similar expression patterns and were detected at low levels in all tissues, indicating that *MtDELLA1* and *MtDELLA2* are involved in many aspects of growth and development. Moreover, *MtDELLA3* had higher expression level in pods compared to that in root and stems (Figure 5A), implying that *MtDELLA3* may play more important roles in pod development. The expression levels of *MtGID1B* and *MtGID1C* were higher in root than those in other tissues (Figure 5B), demonstrating that *MtGID1B* and *MtGID1C* may play a significant role in root development than other tissues. However, *MtGID1A* was widely expressed in all tissues tested (Figure 5B), although at low level, suggesting that it may be involved in multiple developmental processes.

### 2.7. The GA Receptors, MtGID1s, Were Able to Interact with MtDELLA Proteins

**Figure 6:**
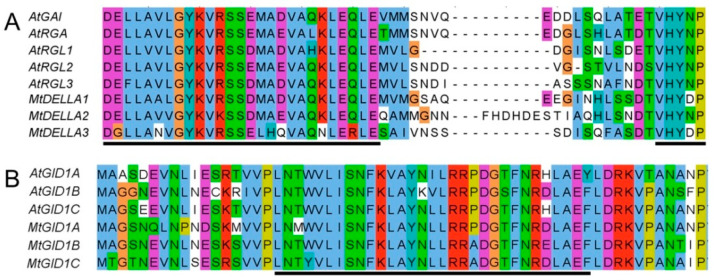
Multiple Sequence alignment of DELLA, VHYNP and SIM domains in DELLA and GID1 proteins. (**A**) DELLA and VHYNP domain sequences alignment for DELLA homologues in *A. thaliana* and *M. truncatula*. Black lines indicate the DELLA (left) and VHYNP (right) domain. (**B**) SUMO-Interaction Motif (SIM) sequences alignment for GID1 homologues in *A. thaliana* and *M. truncatula*. The black line indicates the SIM motif (W[V/I]LI). Amino acids that are conserved throughout are shaded in different colors.

**Figure 7:**
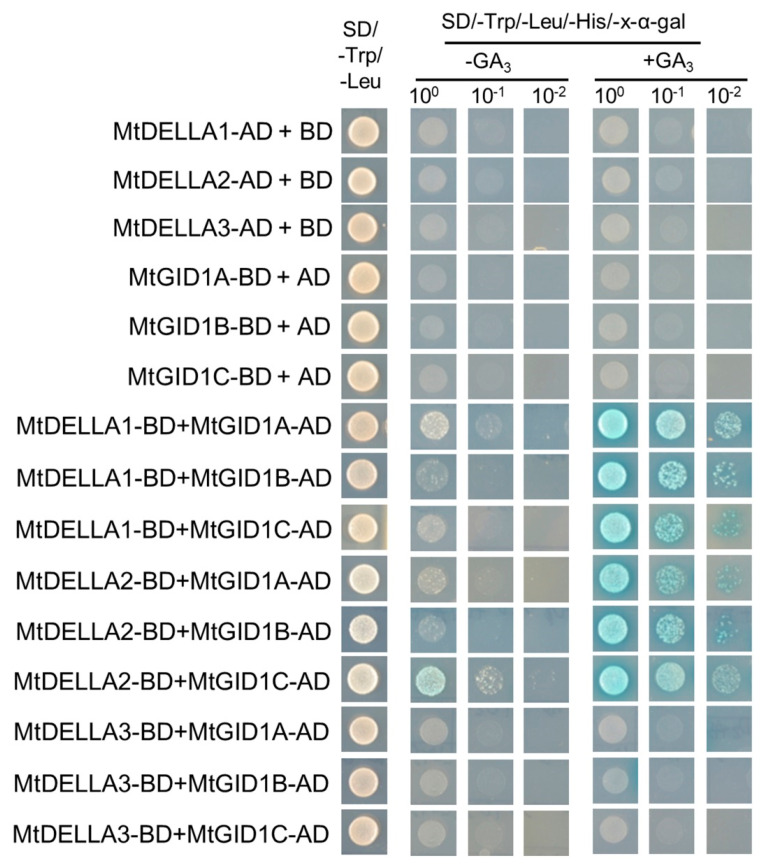
Interaction tests between MtDELLA and MtGID1 proteins in the yeast two-hybrid system. Yeast transformants are spotted onto the control medium (SD/-Leu/-Trp) and selective medium (SD/-Leu/-Trp/-His/-Ade). The initial concentration of the yeast cells spots on SD/-Trp/-Leu (panel 1) and SD/-Trp/-Leu/-His/-Ade medium (panels 2 and 5) were OD600 = 0.2. Then, the yeast cells were diluted 10 and 100 times and were plated onto selective medium (panels 3, 4, 6 and 7) containing 20 μg/mL X-α-gal with or without GA_3_ (10^−5^ M).

In DELLA protein, two important and conserved motifs, DELLA and VHYNP, are involved in the interaction with GID1 proteins [55,56]. In addition, the SUMO-Interaction Motif (SIM), which is important for the recognition of SUMOylated DELLA proteins, is also highly conserved between GID1s [57,58]. Multiple sequences alignment showed that the three MtDELLA proteins had the typical DELLA and VHYNP domains (Figure 6A; Figure S11) and the three MtGID1 receptors had the conserved DELLA interaction SIM motif (Figure 6B; Figure S11). To verify that the MtDELLA and MtGID1 proteins are functional GA signaling components, we assessed the interaction between MtDELLAs and MtGID1s using the yeast two-hybrid system (Figure 7). We also performed the interaction tests between MtDELLAs and MtGID1s in opposite orientation and obtained the same results (Figure S12). MtGID1s interacted with MtDELLA1 and MtDELLA2 in yeast cells in the presence of GA_3_, but not in GA_3_-free medium, while MtGID1s could not interact with MtDELLA3. These results indicate that all three MtGID1s are functional GA receptors and MtDELLA1 and MtDELLA2 may play more important roles in the GA pathway.

### 2.8. The Transcript Level of MtGAoxs Is Regulated by a Feedback Mechanism

**Figure 8:**
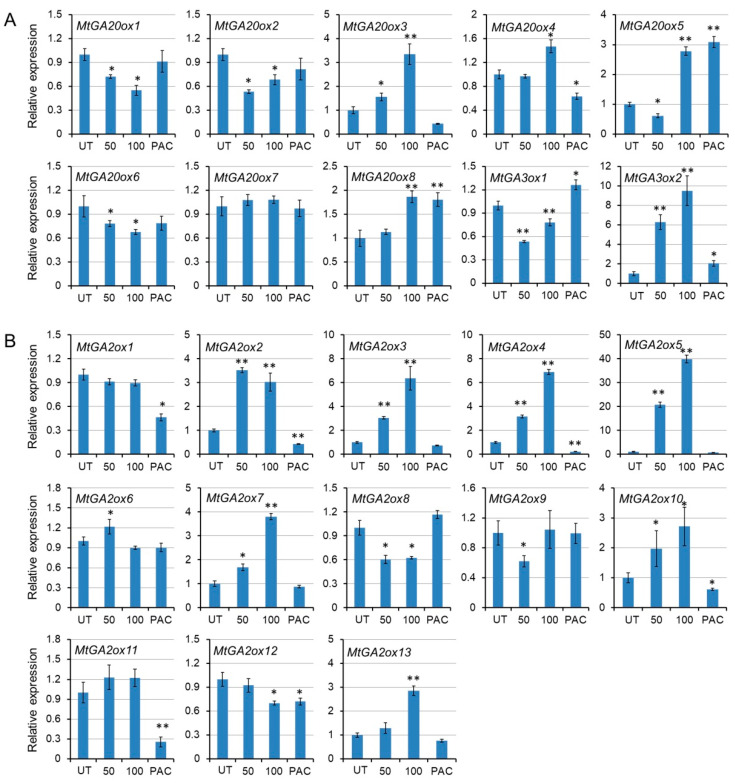
The transcript levels of *MtGA20ox*, *MtGA3ox*, and *MtGA2ox* are regulated by gibberellin 3 (GA_3_) and its inhibitor paclobutrazol (PAC) through a feedback mechanism. (**A**) The transcript levels of *MtGA20ox* and *MtGA3ox* are regulated by GA_3_ and PAC. (**B**) The transcript levels of *MtGA2ox* are regulated by GA_3_ and PAC. Leaves are treated with 50 and 100 μM GA_3_, and 10 μM PAC. Gene expression is normalized to the control untreated (UT) expression level. Data represent the average of three independent experiments ± SD. * *p* < 0.05, ** *p* < 0.01.

Previous studies indicated that feedback regulation controls the concentrations of active GAs in higher plants and the gibberellin metabolism genes also respond to exogenous GA treatment [59,60]. To investigate whether the expression level of *MtGA20ox*, *MtGA3ox*, and *MtGA2ox* are regulated by a feedback mechanism, leaves were treated with GA_3_ and gibberellin inhibitor PAC, and the transcriptional changes were analyzed using qRT-PCR (Figure 8). qRT-PCR results showed that the transcript levels of *MtGA20ox1*, *MtGA20ox2*, *MtGA20ox6* and *MtGA3ox1* were downregulated after 24 h treatment of 50 µM and 100 µM GA_3_ (Figure 8A), indicating that these genes respond quickly to exogenous GA_3_ treatment. On the opposite, the expressions of *MtGA20ox3*, *MtGA20ox4*, *MtGA20ox5*, *MtGA20ox8,* and *MtGA3ox2* were upregulated when treated with 100 µM GA_3_ (Figure 8A), implying that upregulation of these genes may compensate for the downregulation of *MtGA20ox1*, *MtGA20ox2*, *MtGA20ox6,* and *MtGA3ox1* genes to balance the GA level in plant. However, the transcript levels of *MtGA20ox5*, *MtGA20ox8*, *MtGA3ox1,* and *MtGA3ox2* were upregulated when treated with 100 µM PAC (Figure 8A), indicating that these genes may play important roles in GA synthesis. Meanwhile, GA treatment promoted the transcription of some *MtGA2ox* genes, such as *MtGA2ox2*, *MtGA2ox3*, *MtGA2ox4*, *MtGA2ox5*, *MtGA2ox6*, *MtGA2ox7*, *MtGA2ox10,* and *MtGA2ox13* (Figure 8B), demonstrating that these genes may have key roles in GA inactivation. After treatment with PAC, the downregulated genes were *MtGA2ox1*, *MtGA2ox2*, *MtGA2ox4*, *MtGA2ox10*, *MtGA2ox11*, and *MtGA2ox12* (Figure 8B). These data showed that *MtGA20oxs* are responsible for the regulation of bioactive GA synthesis and *MtGA2oxs* regulate the degradation of GA.

### 2.9. GA Has a Positive Effect on Growth and Biomass of M. truncatula and Alfalfa

**Figure 9:**
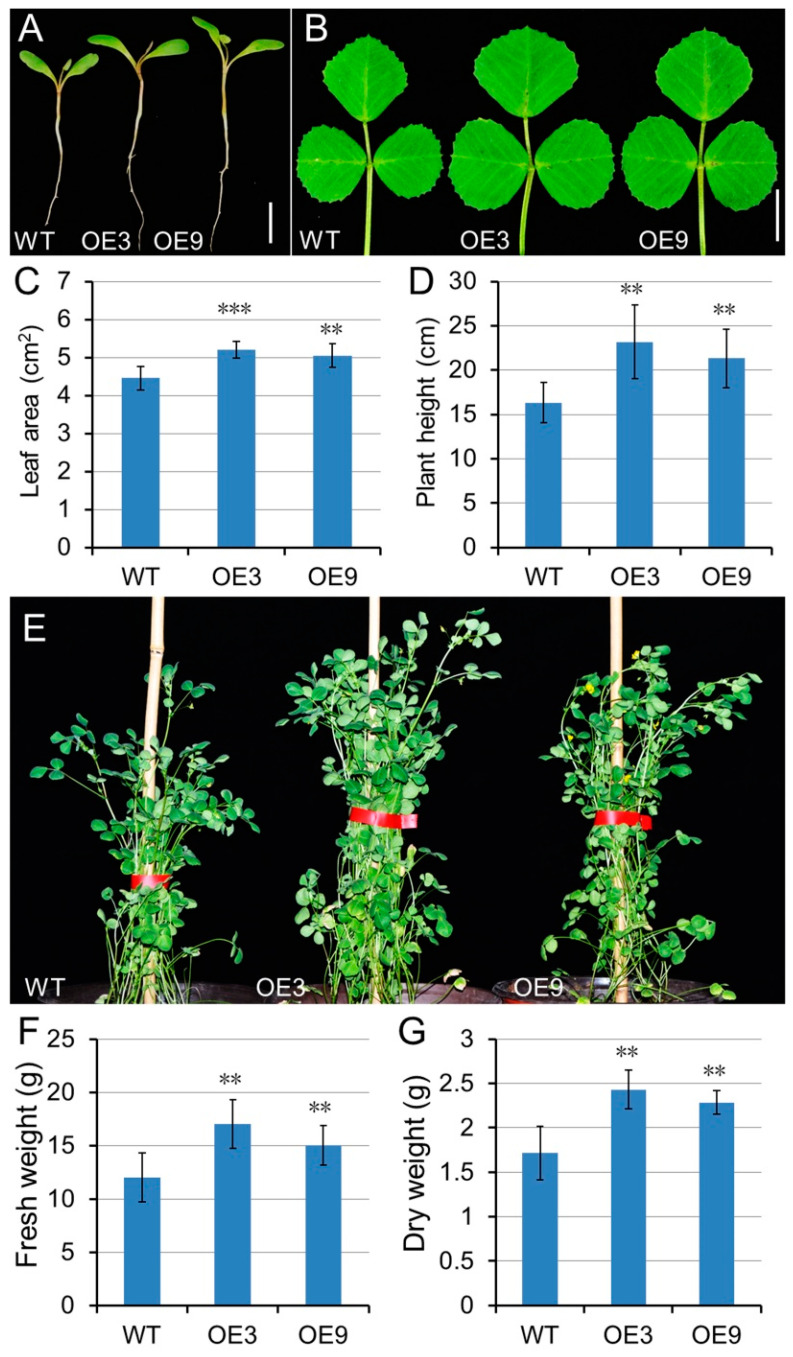
Overexpression of *MtGA20OX1* promoted biomass production. (**A**,**B**) The cotyledeon and leaf phenotypes of the wild type and *MtGA20OX1* transgenic lines. (**C**) Measurements of the blade area of compound leaves on the fifth node of 4-week-old WT and *MtGA20OX1* transgenic plants (means ± SD; *n* = 5). (**D**) The plant height of *MtGA20OX1* transgenic and wild type plants within 50 days (means ± SD; *n* = 13). (**E**) Morphology of wild-type (WT) and *MtGA20OX1* overexpressing *M. truncatula* plants. (**F**,**G**) The fresh and dry weight of developing wild type and *MtGA20OX1* transgenic plants (means ± SD; *n* = 10). Bars = 1 cm in A and B. ** *p* < 0.01, *** *p* < 0.001.

To further investigate the in vivo function of GA, wild-type *Medicago* plants were transformed with *35S:MtGA20OX1* (Figure S13A). Fourteen regeneration lines were obtained and eleven were positive by PCR analysis (Figure S13B). Quantitative real-time PCR data showed that transcripts of *MtGA20OX1* were significantly increased in these transgenic plants, and the overexpression lines (OE) 3 and 9 were selected for further analysis (Figure S12C). To evaluate the effects of *MtGA20OX1* expression on *Medicago* development, the following traits were measured: leaf size, plant height, and biomass. Under the same growth conditions, the *MtGA20OX1* overexpression lines exhibited larger cotyledon and leaf size, compared with those of the controls (Figure 9A–C). Moreover, the plant height of *MtGA20OX1* transgenic lines was measured. The *MtGA20OX1* overexpression lines showed an increase in plant height, compared with that of the controls (Figure 9D–E). Then, we evaluated total above-ground biomass yield of 10 weeks old wild-type and *MtGA20OX1* transgenic plants. The average fresh and dry weight of the *MtGA20OX1* transgenic plants had a significant increase in total biomass, compared with those of wild-type (Figure 9F–G).

### 2.10. Transcription Analysis of Cell Development Related Genes in Transgenic Lines

**Figure 10:**
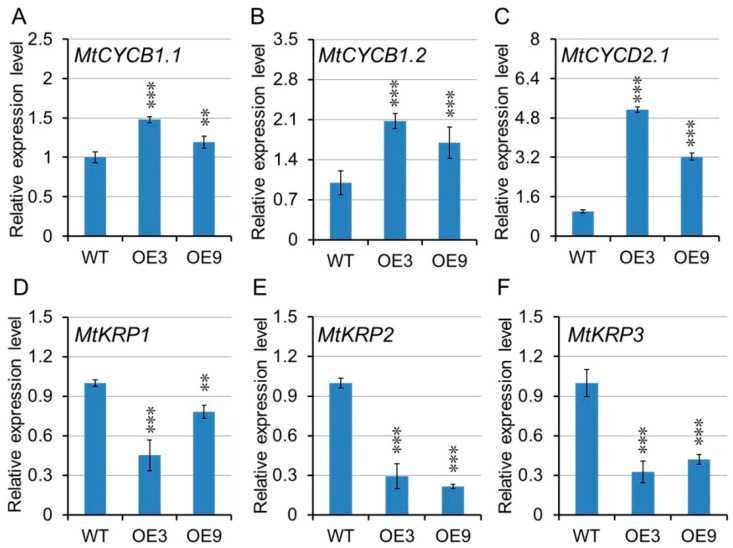
Expression analysis of cell development related genes in wild-type and *MtGA20OX1* transgenic lines. (**A**–**C**) The expression of cyclindependent protein kinase genes, *MtCYCB1.1*, *MtCYCB1.2,* and *MtCYCD2.1*. (**D**–**F**) The expression of *MtKRP1*, *MtKRP2,* and *MtKRP3* genes. ** *p* < 0.01, *** *p* < 0.001.

To explore the molecular mechanism of the increasing biomass of *MtGA20OX1*-overexpressing plants, the expression level of cell division related genes were examined. The results showed that the transcript levels of *MtCYCB1.1*, *MtCYCB1.2,* and *MtCYCD2.1* were up-regulated in *MtGA20OX1*-overexpressing plants (Figure 10A–C), whereas *MtKRP1*, *MtKRP2,* and *MtKRP3* genes encoding the cyclin-dependent kinase inhibitor, were down-regulated (Figure 10D–F). These results suggest that overexpression of *MtGA20OX1* might promote plant growth by controlling cell division.

## 3. Discussion

Gibberellins (GAs) play a crucial role in regulating plant growth and development [9,61,62,63]. However, to date, little was known about the gene families involved in GA biosynthesis, catabolism, and signaling in *M. truncatula*. In this study, 8 *MtGA20ox*, 2 *MtGA3ox*, 13 *MtGA2ox*, 3 *MtGID1*, and 3 *MtDELLA* genes were identified in *M. truncatula* through bioinformatics analysis. Moreover, we evaluated the developmental responses of *Medicago* and alfalfa to GA_3_ and PAC treatment.

The *MtGAox* genes were distributed among chromosomes with different densities. The number of *MtGA20ox* and *MtGA2ox* genes was 1.75-fold larger than that in *Arabidopsis*, suggesting that the *MtGA20ox* and *MtGA2ox* ancestor may have experienced gene expansion and there may be gene redundancies in functions [14,15,64,65]. However, the GA3ox subfamily included 4 AtGA3ox and 2 MtGA3ox proteins, suggesting that the MtGA3ox family have experienced gene loss during evolution compared with that of Arabidopsis. Gene structure analysis indicated that the exon-intron organization of GAox members in the same group were highly conserved in *Medicago*, *Arabidopsis,* and rice [66].

The patterns of gene structure diversity and motif composition can provide significant evidence of the evolutionary relationships of multi-gene families [67,68]. Gene structure and motif analysis revealed that the most closely related members of the MtGAoxs family showed similar exon-intron organization and motif distribution, implying that they is functional conservation in the gibberellin metabolic pathway. Specific motifs in amino acid sequences are important regions for their functions. All of the MtGAox proteins contained the conserved 2OG-FeII_Oxy and DIOX_N domains.

Gene expression patterns analysis can be used to predict the molecular functions of genes in the development of different organs or tissues. In our study, the GA biosynthesis genes, *MtGA3ox2*, *MtGA20ox3*, *MtGA20ox2*, and *MtGA20ox8*, were specifically expressed in root, stem, flower, and pod. Further, additional studies are needed to explore the function of these genes in specific organs in more detail. However, the GA deactivation genes were more widely expressed in all organs, demonstrating that they may play critical roles in processes involved in growth and development of *M. truncatula*. *MtDELLA1*, *MtDELLA2*, and *MtGID1A* were highly expressed in all organs, indicating that these genes might play more important roles in GA signaling transduction in *M. truncatula*.

Previous studies have indicated that the feedback regulation mechanism controls the concentration of active GAs in higher plants [69,70]. In most plants, *GA20ox* and *GA3ox* genes were involved in the final steps of GA biosynthesis and the expression of *GA20ox* and *GA3ox* was downregulated by exogenous GA treatment [59,70]. In contrast, the transcript of *GA2ox* genes was upregulated by GA treatment [65]. In *Vitis Vinifera* L. and *Camellia sinensis* (L.) O. Kuntze, the expression levels of *VvGA20ox1*, *VvGA20ox2*, *CsGA20ox2*, and *CsGA20ox3* were downregulated under GA_3_ treatment and the same was observed for the expression of *MtGA20ox1*, *MtGA20ox2,* and *MtGA3ox1* in *M. truncatula*. In addition, most of the genes encoding MtGA2ox proteins were upregulated by GA_3_ treatment and only two genes, *MtGA2ox2* and *MtGA2ox4*, can respond to both GA_3_ and PAC treatments. Our results demonstrated that the feedback regulation of GA in *M. truncatula* is similar to that of previous studies, indicating that the feedback regulation mechanism is conserved and the *MtGAox* genes are functional components of the GA metabolic pathway [69].

The GID receptors and DELLA proteins are master components of GA signaling [29,31,71]. Phylogenetic analyses showed that the *Medicago* genome contains three GA receptors (MtGID1A, MtGID1B, and MtGID1C) and three DELLA proteins (MtDELLA1, MtDELLA2, and MtDELLA3). The number of MtGID1s is identical to those in *Arabidopsis* and tomato, indicating that the evolutionary mechanisms of MtGID1s are more conservative than MtDELLAs [32,35,57]. In rice, GID1 interacts with DELLA protein SLR1 in a GA-dependent manner in yeast cells [22]. Moreover, AtGID1s can interact with AtDELLA proteins yeast cells in the presence of GA_4_ [33]. In *M. truncatula*, MtGID1s physically interacts with MtDELLA1 and MtDELLA2 in the yeast two-hybrid system in the presence of GA_3_. Although MtDELLA2 and MtGID1C had a weak interaction at the initial concentration (10°) in the absence of GA_3_, further dilution and configuration tests showed that the interaction between MtDELLA2 and MtGID1C is mainly in a GA-dependent manner. These results demonstrate that MtGID1s, MtDELLA1, and MtDELLA2 are functional GA signal components. However, MtGID1s and MtDELLA3 cannot interact with or without GA_3_. Several studies have elucidated that GA regulation of nodulation depends on DELLA proteins in legumes [72,73,74]. It is likely that MtDELLA3 may participate in symbiotic nodulation in a GA-independent manner.

In tomato plants with compound leaves, exogenous GA application resulted in less complex leaves with smooth leaf margins [47,75]. However, leaf complexity is not suppressed by GA treatment in *M. truncatula* and *alfalfa*, whose leaves consist of three leaflets, indicating that the effects of GA on leaf complexity may be species-dependent. Furthermore, GA promotes stem elongation in *M. truncatula* and this function is conserved in multiple species [54,76,77,78,79,80]. It has long been known that GA controls plant growth and development by regulating cell expansion, cell elongation, and cell division. In this study, the *MtGA20OX1*-overexpressing plants showed increased leaf size compared to the wild-type. The cyclin-dependent protein kinase genes were up-regulated in transgenic lines, while, the inhibitor genes were down-regulated. In the future, genetic improvement of alfalfa may be achieved by enhancing GA biosynthesis or signals, such as ectopic expression of *GA20ox* and receptors GID1s or mutation in *GA2oxs* and *DELLA*.

## 4. Materials and Methods

### 4.1. Plant Materials and Treatments

*M. truncatula* wild-type (R108 ecotype) and *35S:MtGA20OX1* seeds were scarified and germinated at 4 °C for one week. The geminated seeds were transferred to nursery seedling plate (4 × 4 × 4 cm Length, Width, Height) for 2 weeks in a light incubator (day: 24 °C, 16 h; night: 22 °C, 8 h; relative humidity: 75%). Then, the seedlings were transferred to pots (15 × 15 × 25 cm Length, Width, Height) with soil mix (soil: vermiculite = 3:1) and grown in a greenhouse at 24 °C (day)/22 °C (night) with 16 h (day)/8 h (night) photoperiod, and relative humidity at 70 to 80%. For GA and paclobutrazol (PAC) treatment, GA_3_ and PAC were dissolved in ethanol at a stock concentration of 100 mM and 10 mM. One-month-old wild-type plants grown in pots were treatment with GA_3_ and PAC at the final concentration of 100 µM and 10 µM for four weeks. Distilled water was used as a control. Each treatment was performed with 20 biological triplicates.

### 4.2. Identification and Phylogenetic Analysis of GA20ox, GA3ox, GA2ox, GID, and DELLA Genes in M. truncatula

To identify the GA metabolism and signaling genes in *M. truncatula* genome, we used 5 AtGA20ox, 4 AtGA3ox, 7 AtGA2ox, 3 AtGID1, and 5 AtDELLA protein sequences to BLAST the *Medicago truncatula* genome sequence (www.medicagogenome.org/). The *Arabidopsis* GA metabolism and signaling genes were obtained from TAIR (www.arabidopsis.org/). Totally, 8 *MtGA20ox*, 2 *MtGA3ox*, 13 *MtGA2ox*, 3 *MtGID1*, and 3 *MtDELLA* genes were identified in *M. truncatula* genome using blast with an E-value <1 × 10^−4^. The protein sequences of these genes were listed in Table S4.

Multiple protein sequences alignment was performed using Jalview 2.10.5 software (www.jalview.org/). The phylogenetic tree of DELLA and GID1 proteins from *A. thaliana* and *M.truncatula* was constructed by MEGA 7.0 using the Neighbor-Joining (NJ) method with the following parameters: Poisson correction, pair-wise deletion and bootstrap values in percentages with 1000 replicates. The phylogenetic tree of GAoxs proteins from *A. thaliana* and *M.truncatula* was constructed on iTOLv5 (Interactive Tree of Life; https://itol.embl.de/) online website.

### 4.3. Chromosome Location, Gene Structure, and Motif Detection

The information, including chromosome length, gene location, and gene length, of *MtGA20ox*, *MtGA3ox*, and *MtGA2ox* genes on the chromosome were obtained from the *M. truncatula* genome database. MapGene (http://mg2c.iask.in/mg2c_v2.1/) was used to visualize the chromosomal distribution of these genes in *M. truncatula* genome.

The exon-intron structures of the *MtGA20ox*, *MtGA3ox*, *MtGA2ox*, *MtGID1*, and *MtDELLA* genes were drawn using the online GSDS 2.0 website (http://gsds.cbi.pku.edu.cn/) by comparing the coding sequences with their corresponding genomic sequences. The conserved protein motifs were analyzed using the MEME online tool (http://meme-suite.org/) with the following parameters: maximum number of motifs of 20 and the optimum width from 6 to 200 residues.

### 4.4. RNA Extraction and qRT-PCR

To detect the relative expression levels of *MtGA20ox*, *MtGA3ox*, *MtGA2ox*, *MtGID1*, and *MtDELLA* genes in root, shoot, leaf, flower, stem, and pod, total RNA was extracted from these tissues on two-month-old wild-type plants. To detect the effects of GA_3_ and PAC on the expression of *MtGA20ox*, *MtGA3ox*, and *MtGA2ox*, total RNA of leaves was extracted from plants after 24 h GA_3_ and PAC treatment. To detect the relative expression levels of *MtGA20ox1*, *MtCYCB*, *MtCYCD*, and *MtKPR*, total RNA of leaves was extracted from two-month-old wild-type and overexpression plants.

Total RNA was extracted using a Plant RNA Kit (TransGene Biotech, Beijing, China) following the manufacturer’s instructions. The concentration and quality of the extracted RNA were evaluated using Nanodrop 2000 Spectrophotometer (Thermo Scientific, USA). Reverse transcription of RNA to cDNA was performed with 1 μg total RNA using an iScript cDNA Synthesis Kit (Bio-Rad, Richmond, CA, USA). qRT-PCR was carried out in triplicate for each sample on a CFX Connect™ Detection System (Bio-Rad, Richmond, CA, USA) using TaKaRa SYBR Premix Kit (TaKaRa, Japan). *MtUBI* gene was used as the internal reference gene. The relative expression levels of the genes were calculated using the 2^-ΔΔCT^ method. Primer sequences used for qRT-PCR analysis were listed in Table S3.

### 4.5. Vector Construction and Plant Transformation

To obtain the *MtGA20OX1* overexpression construction, the 1149 bp CDS sequence was amplified using primer pair MtGA20OX1-F/MtGA20OX1-R. The MtGA20OX1 CDS sequence was transferred into the pB7WG2D vector by Gateway LR reaction (Invitrogen, Carlsbad, CA, USA). Then *35S:MtGA20OX1* destination construct was introduced into *Agrobacterium* strain EHA105. For stable transformation, leaves of wild-type were transformed with EHA105 strain containing the *35S:MtGA20OX1* vector. Primers used are listed in Table S3.

### 4.6. Yeast Two-Hybrid

The yeast two-hybrid assay was performed using the Gold System (Clontech, Mountain View, CA, USA). The coding sequence of MtDELLA1, MtDELLA2, and MtDELLA3 were cloned into the pGADT7 and pGBKT7 vectors, and the coding sequences of MtGID1A, MtGID1B, and MtGID1C were cloned into the pGADT7 and pGBKT7 vectors using the Gateway system. The bait and prey plasmids were co-transformed into yeast strain AH109. For the auxotrophic assay, yeast colonies were inoculated onto SD/-Trp/-Leu and SD/-Trp/-Leu/-His/-Ade plates with or without 10^−5^ M GA_3_ and incubated in the dark at 28°C for 3 days. The initial concentration of the yeast cells spots on both SD/-Trp/-Leu and SD/-Trp/-Leu/-His/-Ade plates were OD600 = 0.2. Then, yeast cells were diluted 10 and 100 times and spotted on SD/-Trp/-Leu/-His/-Ade selective media for detail interaction test analysis. All primers used are listed in Table S3.

### 4.7. Statistical Analysis

Statistical analysis was performed using the SPSS 22.0 statistical software (International Business Machines Corporation, Amonk City, NY, USA). The data relating to expression level and size were subjected to one-way ANOVA of variance followed by a comparison of the means according to a significant difference tested at *p* < 0.05. The data are expressed as the mean ± SE.

## Supplementary Materials

Supplementary Materials can be found at https://www.mdpi.com/1422-0067/21/19/7180/s1.
