# Supplementary material for: Systematic Analysis of Gibberellin Pathway Components in Medicago truncatula Reveals the Potential Application of Gibberellin in Biomass Improvement
Source: Int J Mol Sci. 2020 Sep 29;21(19):7180. doi: 10.3390/ijms21197180 (PMC7582545; doi:10.3390/ijms21197180)
Supplement: Supplementary file 1 [file ijms-21-07180-s001.zip › ijms-912581-suppl for publish/Supplementary figures.pdf]

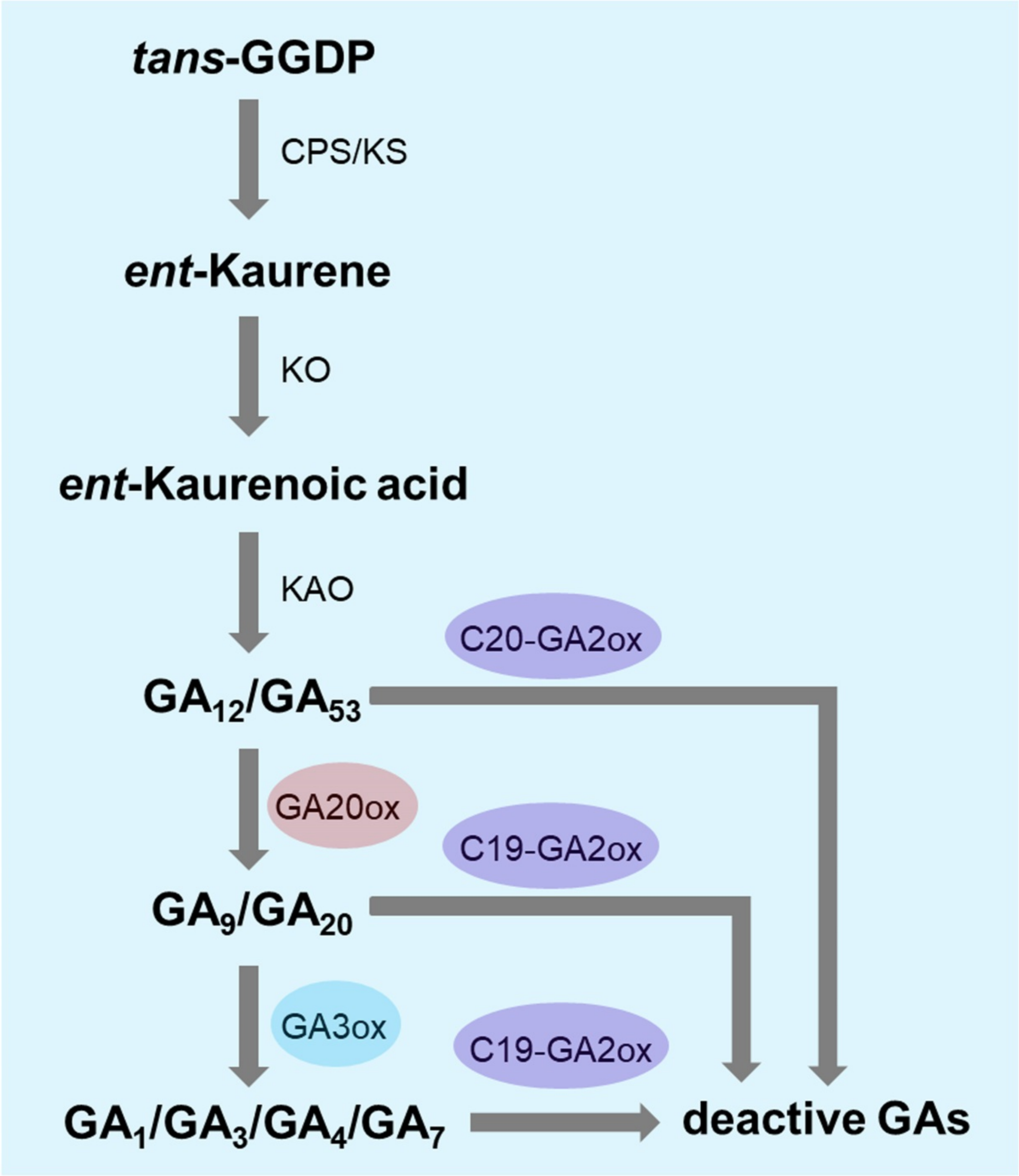

Figure S1. The principal pathways of gibberellin (GA) biosynthesis and deactivation in higher plants. CPS: *ent*-copalyl diphosphate synthase; KS: *ent*-kaurene synthase; KO: *ent*-kaurene oxidase;KAO: *ent*-kaurenoic acid oxidase; GA20ox: GA 20-oxidase; GA3ox: GA 3-oxidase; GA2ox: GA 2-oxidase.

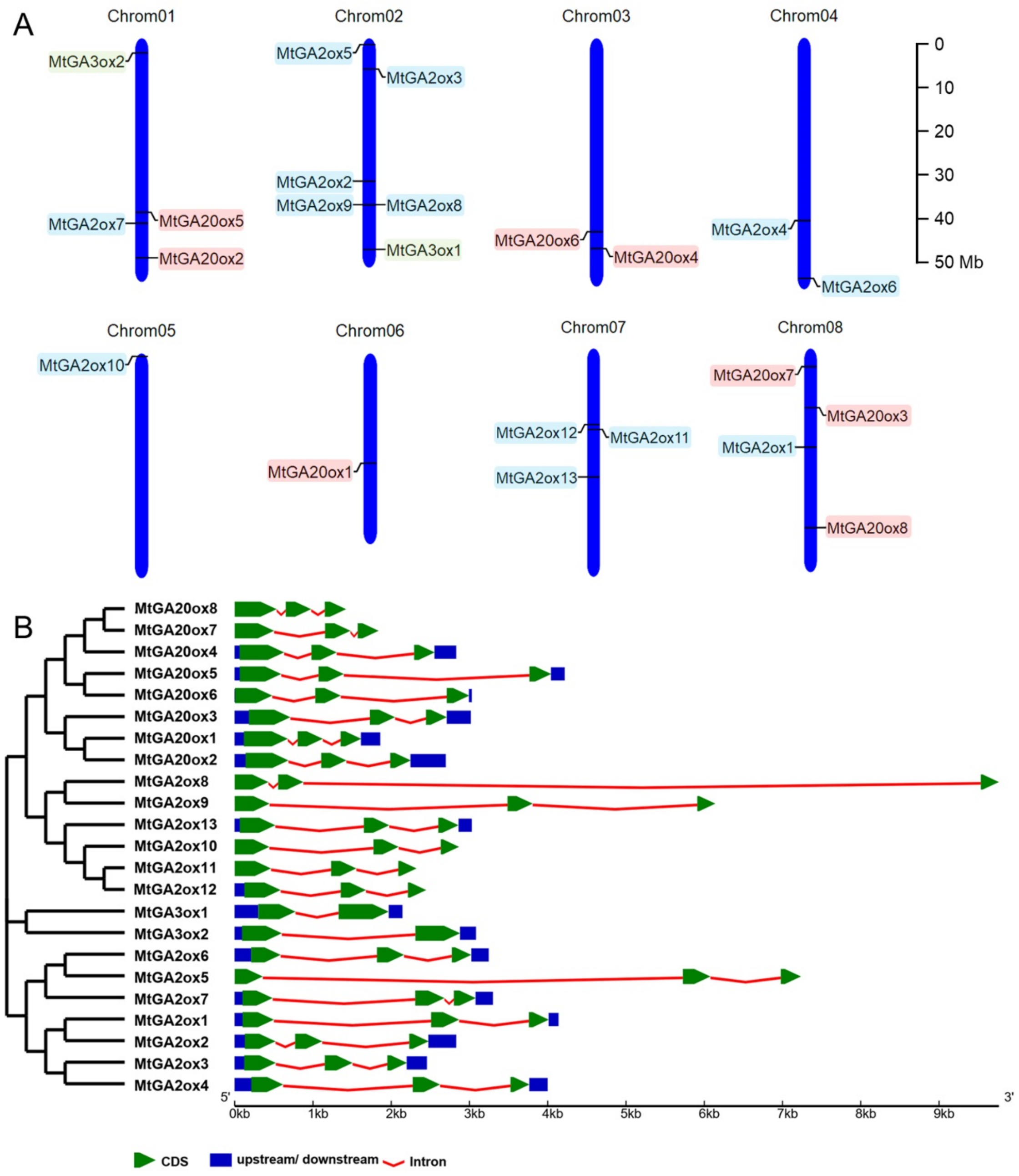

Figure S2. The chromosomal distribution and gene structure analysis of *MtGA20ox*, *MtGA3ox*, and *MtGA2ox* gene family in *M. truncatula*. (A) Distribution of *MtGA20ox*, *MtGA3ox*, and *MtGA2ox* genes in the *M. truncatula* genome. The chromosome number is indicated at the top of each chromosome. The scale is in megabases (Mb). (B) Genomic organization of *MtGA20ox*, *MtGA3ox*, and *MtGA2ox* genes in the *M. truncatula*. Exons are represented by green boxes; introns are shown as red lines; blue boxes represent upstream/downstream regions.

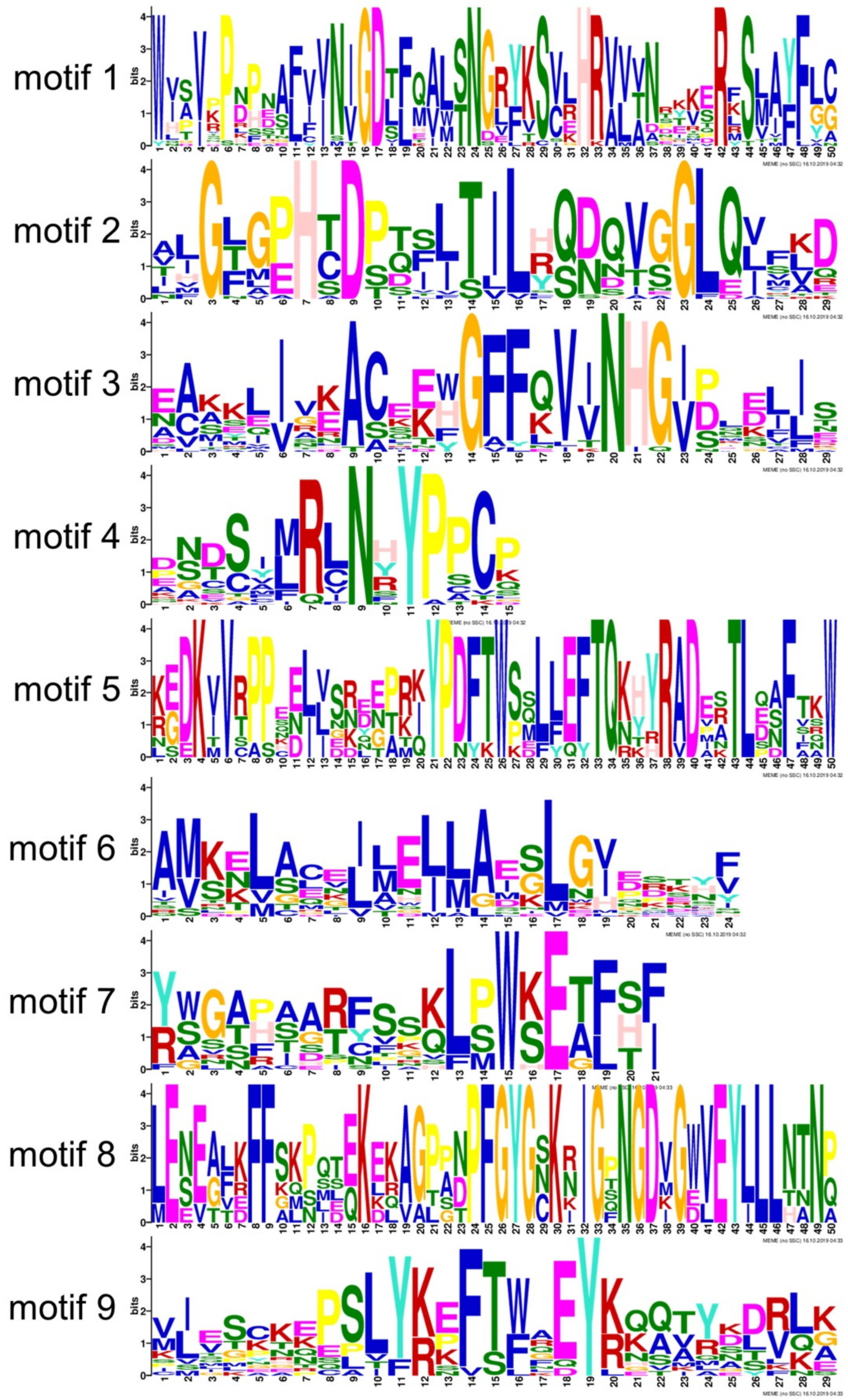

Figure S3. The sequences and logos of the nine identified motifs in MtGA20ox, MtGA3ox, and MtGA2ox proteins.

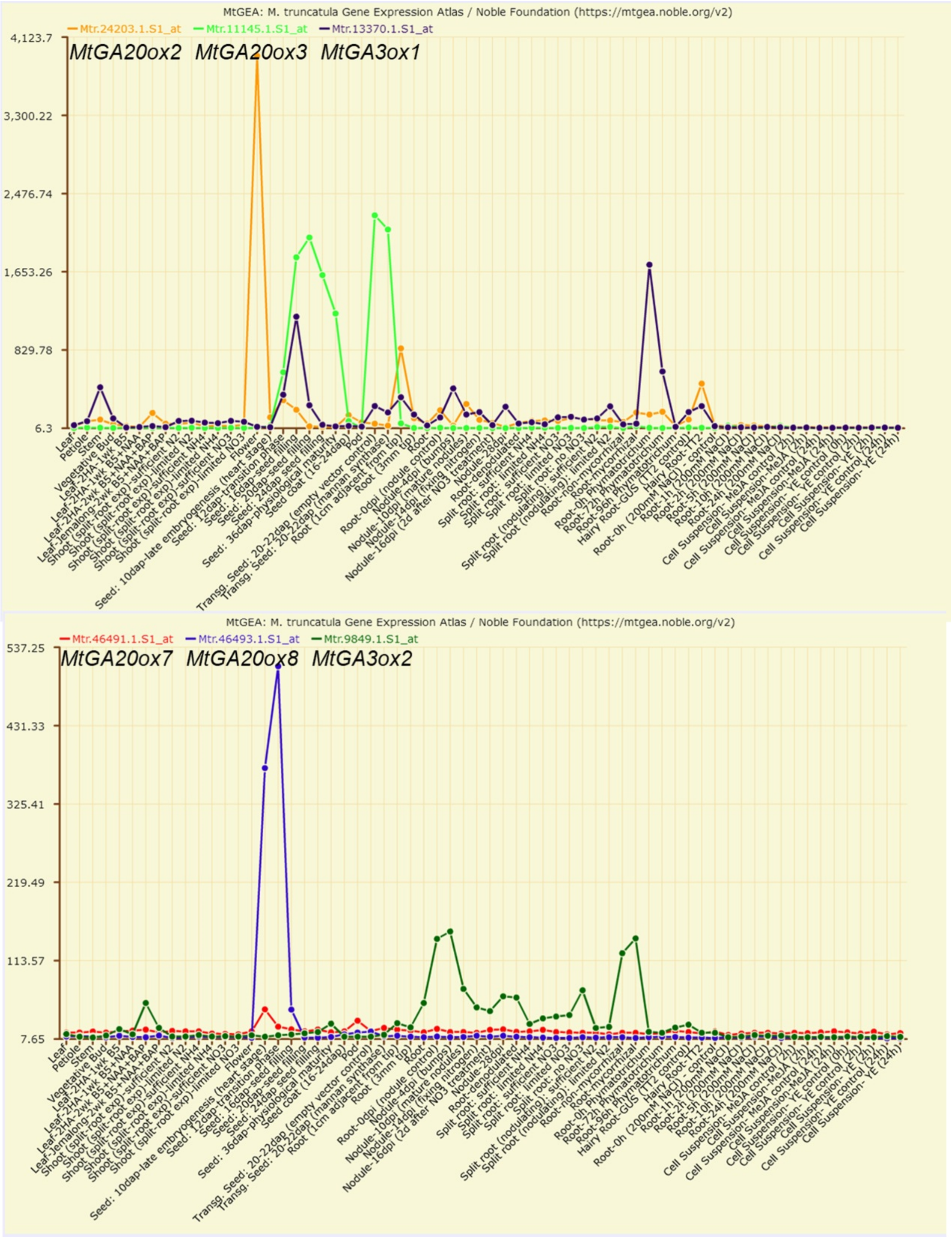

Figure S4. Expression profiling of partial *MtGA20oxs* and *MtGA3oxs* transcripts based on the probe. The data for *Mt20ox2* (probe set Mtr.24203.1.S1_at), *Mt20ox3* (probe set Mtr.11145.1.S1_at), *Mt20ox7* (probe set Mtr.46491.1.S1_at), *Mt20ox8* (probe set Mtr.46493.1.S1_at), *MtGA3ox1* (probe set Mtr.13370.1.S1_at), and *MtGA3ox2* (probe set Mtr.9849.1.S1_at) are accessible at http://bioinfo.noble.org/gene-atlas/.

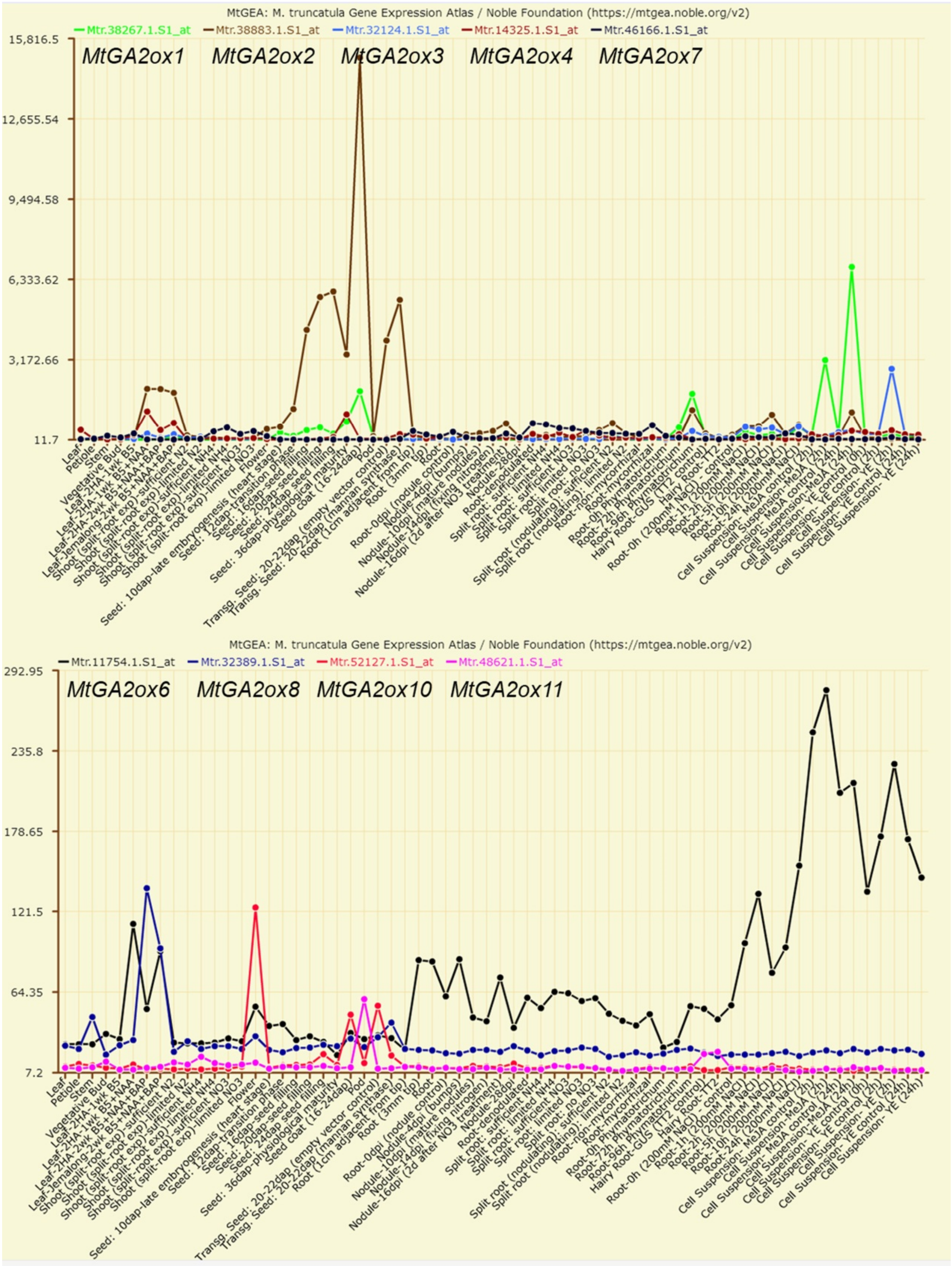

Figure S5. Expression profiling of partial *MtGA2oxs* transcripts based on the probe. The data for *Mt2ox1* (probe set Mtr.38267.1.S1_at), *Mt2ox2* (probe set Mtr.38883.1.S1_at), *Mt2ox3* (probe set Mtr.32124.1.S1_at), *Mt2ox4* (probe set Mtr.14325.1.S1_at), *MtGA2ox6* (probe set Mtr.11754.1.S1_at), *MtGA2ox7* (probe set Mtr.46166.1.S1_at), *MtGA2ox8* (probe set Mtr.32389.1.S1), *MtGA2ox10* (probe set Mtr.52127.1.S1), and *MtGA2ox11* (probe set Mtr.48621.1.S1_at) are accessible at http://bioinfo.noble.org/gene-atlas/.

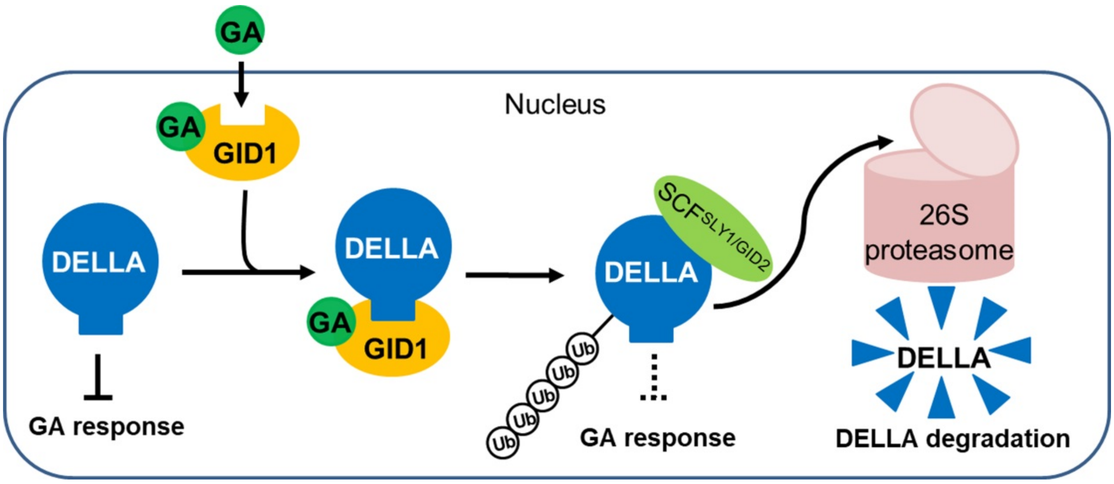

Figure S6. Model of the interactions of the established GA signaling components. Bioactive GA, when bound to the GID1 receptor, induces the interaction of the GID1 with the DELLA proteins. This event is followed by DELLA protein ubiquitylation via $SCF^{SLY1/GID2}$ and DELLA protein degradation by the 26S proteasome.

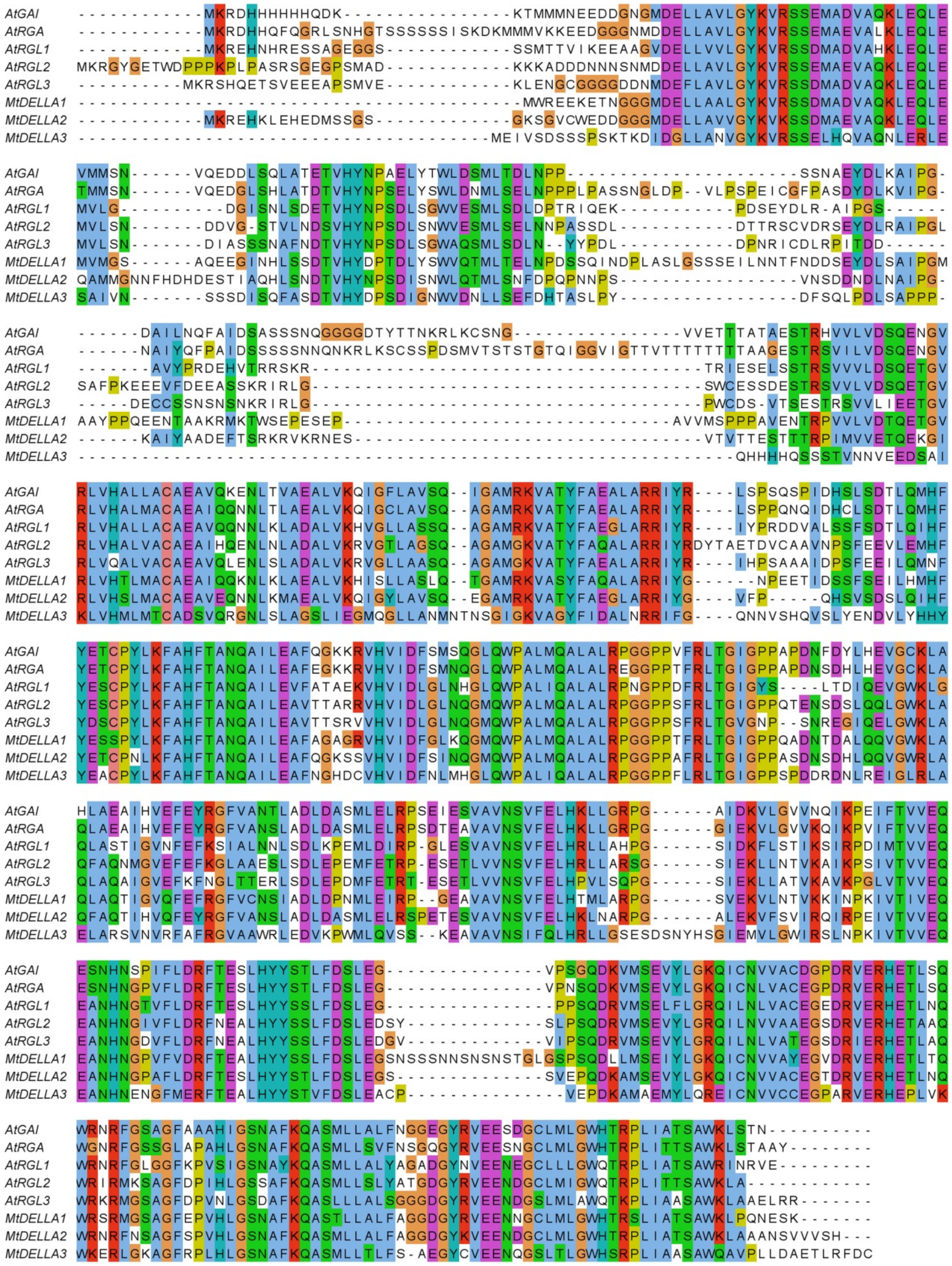

Figure S7. Sequence alignment of DELLA proteins from *M. truncatula* and *A. thaliana*. Amino acids that are conserved throughout are shaded in different colors.

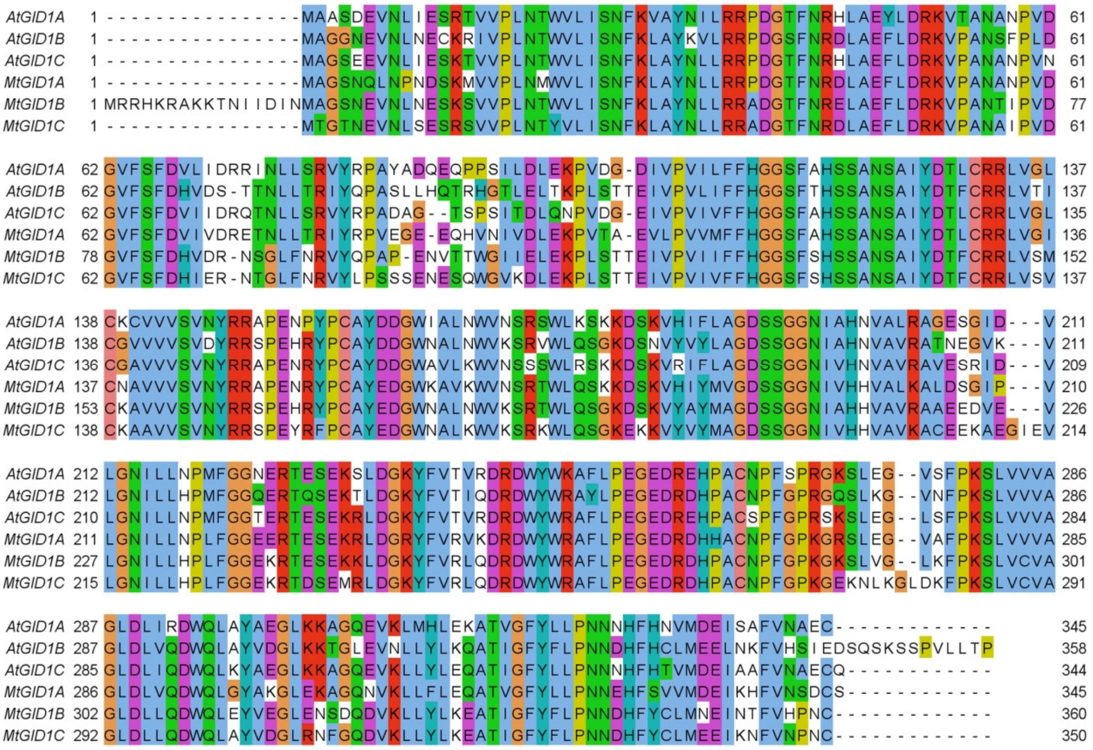

Figure S8. Sequence alignment of GID1 proteins from *M. truncatula* and *A. thaliana*. Amino acids that are conserved throughout are shaded in different colors.

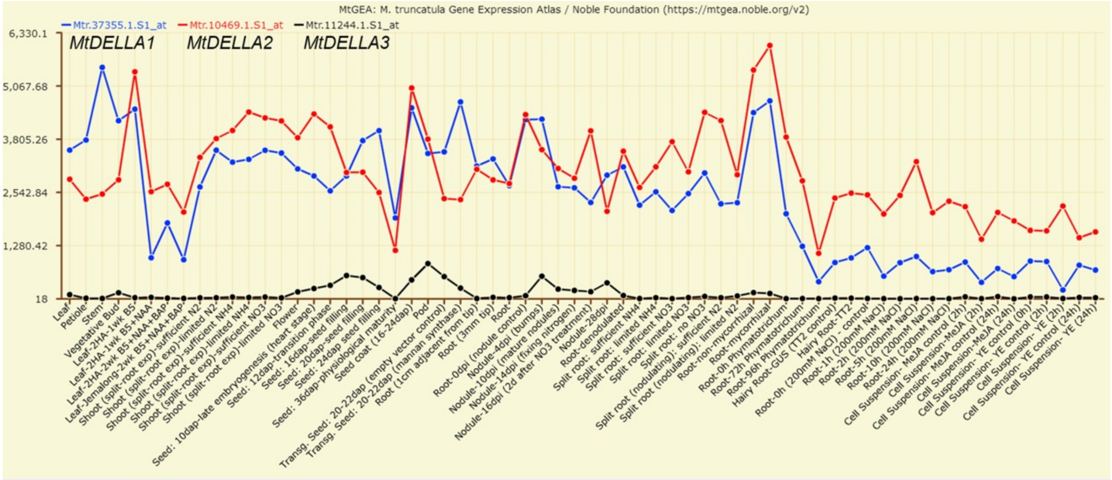

Figure S9. Expression profiling of *MtDELLA1*, *MtDELLA2*, and *MtDELLA3* transcripts based on the probe. The data for *MtDELLA1* (probe set Mtr.37355.1.S1_at), *MtDELLA2* (probe set Mtr.10469.1.S1_at), and *MtDELLA3* (probe set Mtr.11244.1.S1_at) are accessible at http://bioinfo.noble.org/gene-atlas/.

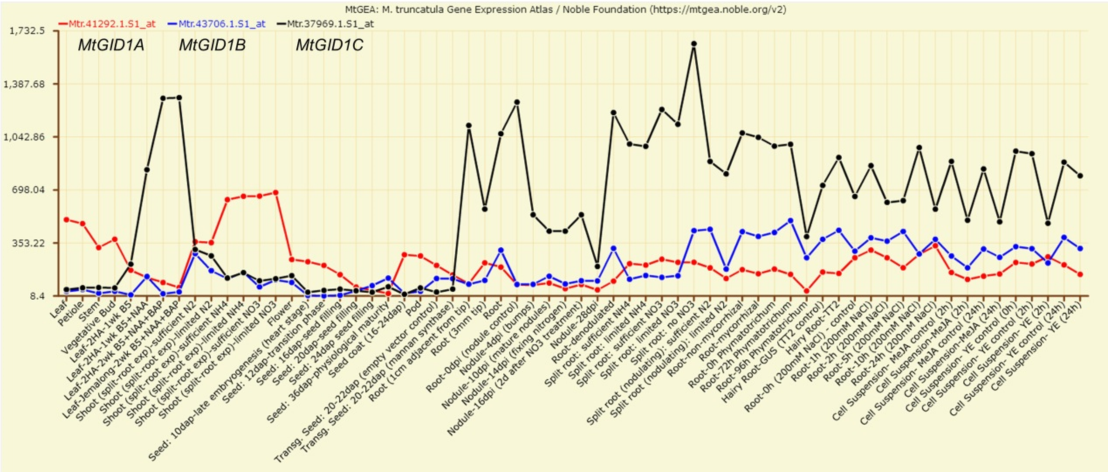

Figure S10. Expression profiling of *MtGID1A*, *MtGID1B*, and *MtGID1C* transcripts based on the probe. The data for *MtGID1A* (probe set Mtr.41292.1.S1_at), *MtGID1B* (probe set Mtr.43706.1.S1_at), and *MtGID1C* (probe set Mtr.37969.1.S1_at) are accessible at http://bioinfo.noble.org/gene-atlas/.

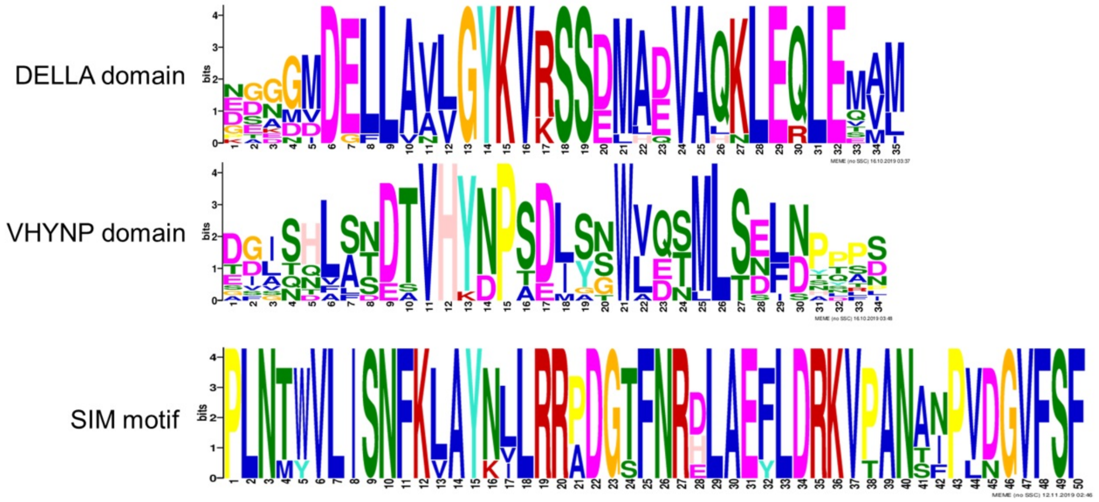

Figure S11. The sequences and logos of the DELLA and VHYNP domain in DELLA proteins and SIM motif in MtGID1 proteins.

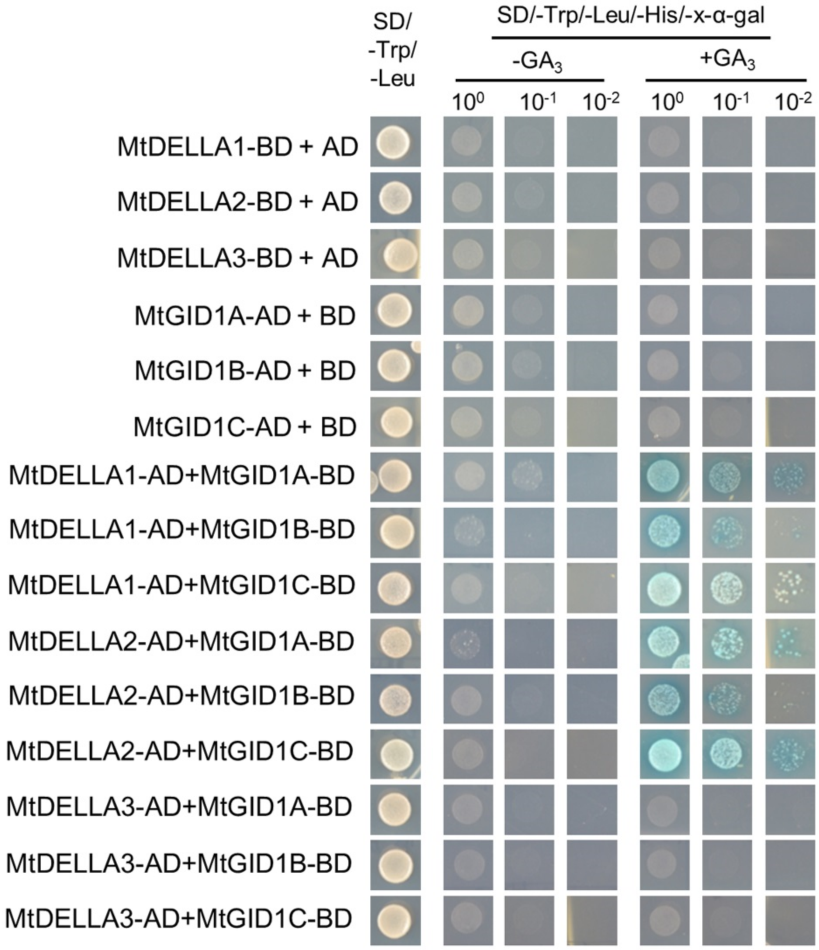

Figure S12. Interaction tests between MtDELLA and MtGID1 proteins in the yeast two-hybrid system. Yeast transformants are spotted onto control medium (SD/-Leu/-Trp) and selective medium (SD/-Leu/-Trp/-His/-Ade). The initial concentration of the yeast cells spots on SD/-Trp/-Leu and SD/-Trp/-Leu/-His/-Ade medium (panels 1, 2 and 5) were OD600=0.2. Then, the yeast cells were diluted 10 and 100 times and were plated onto selective medium (panels 3, 4, 6 and 7) containing 20 μg/mL X-α-gal with or without GA_3 (10^{-5} M).

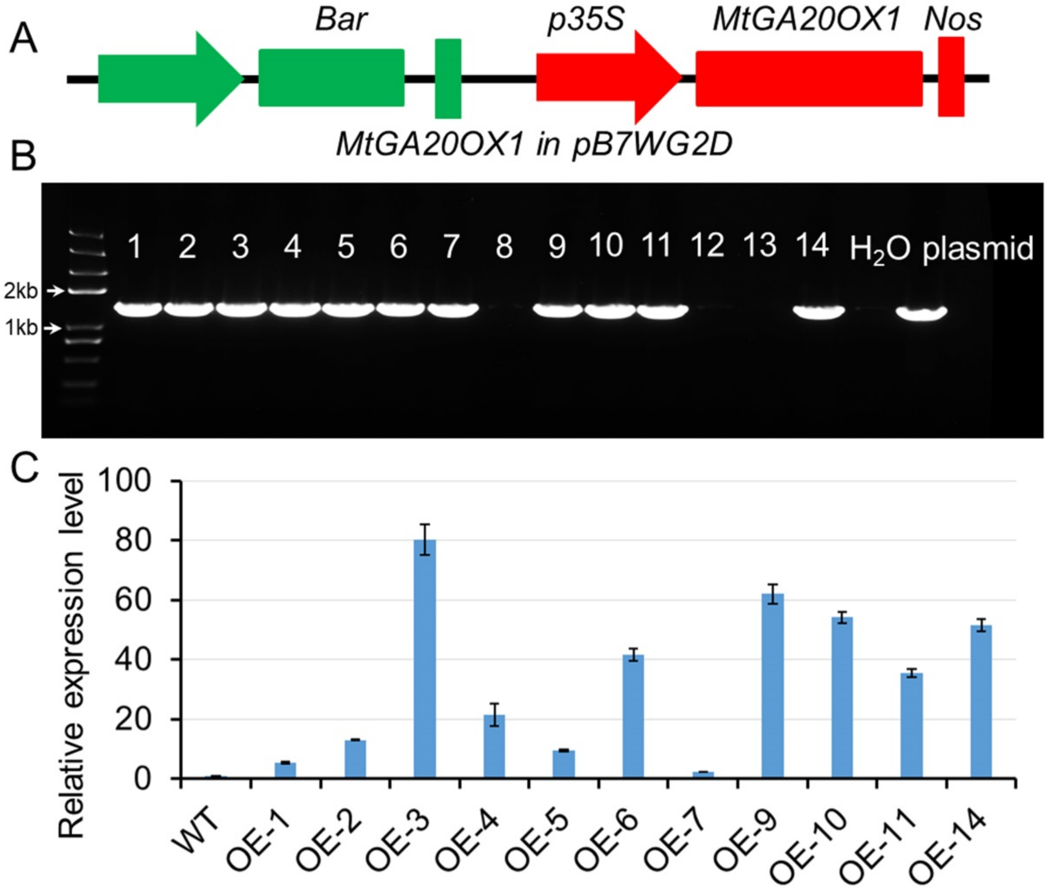

Figure S13. Molecular characterization of *Medicago MtGA20OX1* overexpression lines. **(A)** Schematic illustration of vector used for *MtGA20OX1* overexpression. **(B)** PCR analysis of regenerated transgenic plants together with the positive control (35S-MtGA20OX1-Nos plasmid) and negative control (H_2O). The amplified fragments in transgenic lines and positive control were 1206bp. **(C)** Transcript levels of *MtGA20OX1* in wild type and *35S: MtGA20OX1* overexpressing plants (OE).
