# Supplementary material for: Systematic Analysis of Gibberellin Pathway Components in Medicago truncatula Reveals the Potential Application of Gibberellin in Biomass Improvement
Source: Int J Mol Sci. 2020 Sep 29;21(19):7180. doi: 10.3390/ijms21197180 (PMC7582545; doi:10.3390/ijms21197180)
Supplement: Supplementary file 1 [file ijms-21-07180-s001.zip › ijms-912581-suppl for publish/Supplementary Tables.pdf]

Table S1. Genome-wide identification of *MtGAox* genes in *M. truncatula*.

Gene Name	Gene Accession	Location	CDS (bp)	Exon	Peptide (aa)
MtGA20ox1	*Medtr6g464620*	chr6:22552607..22554465-	1149	3	382
MtGA20ox2	*Medtr1g102070*	chr1:46102116..46104813+	1134	3	377
MtGA20ox3	*Medtr8g033380*	chr8:12871549..12874562-	1119	3	372
MtGA20ox4	*Medtr3g096500*	chr3:44112343..44115170-	1155	3	384
MtGA20ox5	*Medtr1g081840*	chr1:36441803..36446020+	1131	3	376
MtGA20ox6	*Medtr3g088745*	chr3:40552583..40555611+	1089	3	362
MtGA20ox7	*Medtr8g093930*	chr8:39305771..39307609-	1095	3	364
MtGA20ox8	*Medtr8g093980*	chr8:39314676..39316099-	1134	3	377
MtGA3ox1	*Medtr2g102570*	chr2:44198670..44200810+	1116	2	371
MtGA3ox2	*Medtr1g011580*	chr1:2083698..2086776+	1080	2	359
MtGA2ox1	*Medtr8g461330*	chr8:21569107..21573242+	1014	3	337
MtGA2ox2	*Medtr2g070870*	chr2:29875542..29878373-	987	3	328
MtGA2ox3	*Medtr2g019370*	chr2:6312834..6315290-	1011	3	336
MtGA2ox4	*Medtr4g096840*	chr4:38542890..38546885+	999	3	332
MtGA2ox5	*Medtr2g033270*	chr2:12609171..12616406-	969	3	322
MtGA2ox6	*Medtr4g123020*	chr4:50754413..50757657+	972	3	321
MtGA2ox7	*Medtr1g086550*	chr1:38725663..38728964-	1038	3	345
MtGA2ox8	*Medtr2g083000*	chr2:34818539..34828303+	984	3	327
MtGA2ox9	*Medtr2g083030*	chr2:34841919..34848060-	999	3	332
MtGA2ox10	*Medtr5g005570*	chr5:505257..508123-	999	3	332
MtGA2ox11	*Medtr7g451860*	chr7:17676319..17678642-	1020	3	339
MtGA2ox12	*Medtr7g047670*	chr7:16702083..16707043-	1020	3	339
MtGA2ox13	*Medtr4g074130*	chr4:28190446..28193469+	1038	3	345

Table S2. Genome-wide identification of *MtDELLA* and *MtGID* genes in *M. truncatula*.

Gene Name	Gene Accession	Location	CDS (bp)	Exon	Peptide (aa)
MtGID1A	*Medtr1g082210*	chr1:36556311..36558884+	1038	2	345
MtGID1B	*Medtr1g089310*	chr1:40066098..40068858+	1083	3	360
MtGID1C	*Medtr7g093950*	chr7:37385381..37387711+	1053	2	350
MtDELLA1	*Medtr3g065980*	chr3:29797559..29799202-	1644	1	547
MtDELLA2	*contig_52215*	unknown	1602	1	533
MtDELLA3	*contig_55897*	unknown	1506	1	501

Table S3. Primers used in this study.

MtGA20ox1-F	ATGGCTATAGAGTGCATAACAAGTA	For clone of *MtGA20ox1* gene CDS
MtGA20ox1-R	ACTTTTCTAATAGGGTTCTCAATGG	
MtGA20ox1-qRT-F	GGTGACACTTTCATGGCTCTTTC	For qRT-PCR analysis of *MtGA20ox1*
MtGA20ox1-qRT-R	TTGTTCACCACTGCCCTATGTAA	
MtGA20ox2-qRT-F	TCACTCTAGGCACTGGACCTCAT	For qRT-PCR analysis of *MtGA20ox2*
MtGA20ox2-qRT-R	CCACCTACTTGATCCTGGTGAAG	
MtGA20ox3-qRT-F	AGGCAATTTGGGAGTGTATACCA	For qRT-PCR analysis of *MtGA20ox3*
MtGA20ox3-qRT-R	CCCAAGAAGTTCCATGATTCCA	
MtGA20ox4-qRT-F	TTGCCTCCACAGGGCATTAG	For qRT-PCR analysis of *MtGA20ox4*
MtGA20ox4-qRT-R	GGGGGTCTCACCACTTTGTC	
MtGA20ox5-qRT-F	GGAGGGCTTGATGTGTTTGC	For qRT-PCR analysis of *MtGA20ox5*
MtGA20ox5-qRT-R	CAAATGCATCTGAACGAGGTCTA	
MtGA20ox6-qRT-F	GCTCATGAAGAGCTCCAAGCA	For qRT-PCR analysis of *MtGA20ox6*
MtGA20ox6-qRT-R	TCATTGTCCCCTTTGAGAAACC	
MtGA20ox7-qRT-F	GGTGCAACTCTTACCCACCT	For qRT-PCR analysis of *MtGA20ox7*
MtGA20ox7-qRT-R	TGGGTCACAATGAGGACCAG	
MtGA20ox8-qRT-F	CTTCATGGCACTGACCAATGG	For qRT-PCR analysis of *MtGA20ox8*
MtGA20ox8-qRT-R	CCAGCTCGTTGCTTACCAAAAC	
MtGA3ox1-qRT-F	TCACCCTTCCCTCTCACCAA	For qRT-PCR analysis of *MtGA3ox1*
MtGA3ox1-qRT-R	CCATAGCCCGAAACACCATCT	
MtGA3ox2-qRT-F	TTGGAGCAATGCAACTAAACTTCT	For qRT-PCR analysis of *MtGA3ox2*
MtGA3ox2-qRT-R	GCCAGACCCATTGCTTTTTTT	
MtGA2ox1-qRT-F	AAATCACTACGCGGCATGTG	For qRT-PCR analysis of *MtGA2ox1*
MtGA2ox1-qRT-R	TGCTCCCCAAATCCAATCA	
MtGA2ox2-qRT-F	AGACCCGGAAGCAAAAACTCT	For qRT-PCR analysis of *MtGA2ox2*
MtGA2ox2-qRT-R	CCATGGTTCACCACTTTGAAGA	
MtGA2ox3-qRT-F	TCCCTTCTGATCACAATTCCTTCT	For qRT-PCR analysis of *MtGA2ox3*
MtGA2ox3-qRT-R	GAATCGTCCATTAGTCATAACCTGAA	
MtGA2ox4-qRT-F	GGAACAACATGCAAGCAACTACA	For qRT-PCR analysis of *MtGA2ox4*
MtGA2ox4-qRT-R	TGCATCTGGTTTTGATAGGTCAA	
MtGA2ox5-qRT-F	CCCTTTACATGGGCCCAAT	For qRT-PCR analysis of *MtGA2ox5*
MtGA2ox5-qRT-R	GACGTGAATCTCCCAATCGAA	
MtGA2ox6-qRT-F	GCTCCACCTCTAAATGCTTCCA	For qRT-PCR analysis of *MtGA2ox6*
MtGA2ox6-qRT-R	AGAGGGCCTGGTTGGTGTAA	
MtGA2ox7-qRT-F	TTGCTCCCTCAGTTATGGTTACAC	For qRT-PCR analysis of *MtGA2ox7*
MtGA2ox7-qRT-R	TGCCCAAGTGAATGGTTTGA	
MtGA2ox8-qRT-F	CGAGTGGTAGCTGCTGAGAAAG	For qRT-PCR analysis of *MtGA2ox8*
MtGA2ox8-qRT-R	GCATCAATGGAAGGGCCATA	
MtGA2ox9-qRT-F	TTGGCCTCTTGGCTCATTGT	For qRT-PCR analysis of *MtGA2ox9*
MtGA2ox9-qRT-R	CAATCCCCCGACACTATCTTGA	
MtGA2ox10-qRT-F	CTGAACAAAACCAAAAACAATCGT	For qRT-PCR analysis of *MtGA2ox10*
MtGA2ox10-qRT-R	CCTCCACCACCGGAAACTT	

MtGA2ox11-qRT-F<br>MtGA2ox11-qRT-R	CTGAACAAAACCAAAAACAATCGT<br>CCTCCACCACCGGAAACTT	For qRT-PCR analysis of *MtGA2ox11*
MtGA2ox12-qRT-F<br>MtGA2ox12-qRT-R	GGCTTGGAGCAATGGAGTGT<br>CCTTTCTACTCTTGGGTTTGTCACA	For qRT-PCR analysis of *MtGA2ox12*
MtGA2ox13-qRT-F<br>MtGA2ox13-qRT-R	TTTGCAGCAGCTATGCTAGAAGTC<br>GTGGCCCAAGTTTTCTGCTAGA	For qRT-PCR analysis of *MtGA2ox13*
MtGID1A-qRT-F<br>MtGID1A-qRT-R	CCCAATGATTCAAAGATGGTGG<br>AAAGTTCCATCAGGACGACG	For qRT-PCR analysis of *MtGID1A*
MtGID1A-F	ATGGCTGGAAGTAACCAACTC	For clone of *MtGID1A* CDS
MtGID1A-R	CTATGAACAGTCAGAGTTGACAAAA	
MtGID1B-qRT-F<br>MtGID1B-qRT-R	TCATCACGTTGCGGTGAGAG<br>CCGCCAAAGAGTGGATGGAG	For qRT-PCR analysis of *MtGID1B*
MtGID1B-F	ATGAGGCGGCATAAAAGAGCGAAG	For clone of *MtGID1B* CDS
MtGID1B-R	TCAGCAGTTAGGATGCACAAAGGT	
MtGID1C-qRT-F<br>MtGID1C-qRT-R	CAGAAAAGTCCCCGCAAACG<br>AGACCGGTGTTTCGTTCGAT	For qRT-PCR analysis of *MtGID1C*
MtGID1C-F	ATGACTGGTACTAATGAAGTCAAC	For clone of *MtGID1C* CDS
MtGID1C-R	TTAACAGTTAGGATTAACAAAGTTC	
MtDELLA1-qRT-F<br>MtDELLA1-qRT-R	GCGGACAATACCGATGCTTT<br>CACCGATTGTCTGAGCAAGCT	For qRT-PCR analysis of *MtDELLA1*
MtDELLA1-F	ATGTGGAGAGAAGAAAAAGAAACCAA	For clone of *MtDELLA1* CDS
MtDELLA1-R	TCACTTGGACTCATTTTGTGGAAGC	
MtDELLA2-qRT-F<br>MtDELLA2-qRT-R	TCCACCATCGCTCAACATCTT<br>CAGTTAGAGATATCAGCGGGATTG	For qRT-PCR analysis of *MtDELLA2*
MtDELLA2-F	ATGAAGAGAGAGCATAAGCTTGAAC	For clone of *MtDELLA2* CDS
MtDELLA2-R	TCAGTGCGAAACCACCACTGA	
MtDELLA3 -qRT-F<br>MtDELLA3-qRT-R	TGGCTGGCTCGCTAATCG<br>TGCCGGAATTTGTGTTCATGT	For qRT-PCR analysis of *MtDELLA3*
MtDELLA3-F	ATGGAAATAGTTTCAGATTCTTCTTCTC	For clone of *MtDELLA3* CDS
MtDELLA3-R	TCAACAATCAAAACACAGTGTTTCAGC	
MtUBI-qRT-F<br>MtUBI-qRT-R	CTGACAGCCCACTGAATTGTGA<br>TTTTGGCATTGCTGCAAGC	For qRT-PCR analysis of *MtUBI*

Table S4. The sequences of all genes involved in this study.

>MtGA20ox1
MAIECITSTKAMTQSPKQNHGKNEDEESSLVFDASFLRHTINLPKQFIWPDEEKPCMNVPELDVPLI
DLKNFLSGDPFAAMEASKIIGEACEKHGFFLVVNHGIDAKLIEHAHSYMDGFFENPLSQKQRAQRK
IGEHCGYASSFTGRFSSKLPWKETLSFQFSDEKNSPNIVKDYLCNTLGEDFEEFGEVYQKYCEAMS
TLSLGIMELLGMSLGVGKDCFRDFFEENKSIMRLNYYPPCQKPDLTLGTGPHCDPTSLTILHQDQV
GGLQVFVDNEWHSIRPNFNAFVVNIGDTFMALSNGRYKSCLHRAVVNNKTTRKSLAFFLCPKGDK
VVCPPSELVSDLTPRIYPDFTWPMLLEFTQKYYRADMRTLEAFTKWIQQKSS
>MtGA20ox2
MAIECITSMPQQLLNQETKEQEKPLVFDASVLKHQVNLPTQFIWPDEEQACLNVPELHVPFIDLGGF
LSGDPVAAMEASKVVGEACKKHGFFLVVNHGIDEKLISDAHAFMDDFFELPLSQKQRAQRKTGEH
CGYASSFTGRFSSKLPWKETLSFQFSADEKSPNLVRDYLCNTMGNEFEKFGEVYQDYCKAMSNLS
LGIMEILGMSLGVGKAHFREFFEENSSIMRLNYYPTCQKPELTLGTGPHCDPTSLTILHQDQVGGLQ
VYVDDQWHSISPHFNAFVVNIGDTFMALSNGRYKSCLHRAVVNSEKTRKSLAFFLCPLSDKVVTPP
CELVDNYNPRIYPDFTWSMLLEFTQKHYRADIKTLEAFAKWVQCKST
>MtGA20ox3
MHILNPSMLFAPPNTKEKQCHDTSFLPCQVTNIPSEFIWPEHEKPCLTPPKLQVPPIDLKAFLSGDPK
AISNACSQVNDACKKHGFFLVVNHGVDKKLLAQAHKLVDDFFCMQLCEKQKAQRKVGEHCGYA
NSFIGRFSSKLPWKETLSFRYSDDKSCRTVEDYFVNVMGEDFRQFGSVYQDYCEAMSNLSLGIMEL
LGMSLGVDKEYFRHFFEANDSVMRLNYYPPCKNPDLALGTGPHCDPTSLTILHQDQVEGLQVLVD
GIWHSIVPKEDAFVVNIGDTFMALSNGIFKSCLHRAVVNDTIVRKSLAFFLCPNEEKIVTPPKELINK
ENPMIYPNFTWPSLLEFTQKHYRADERTLDAFSRWLQEKQLN
>MtGA20ox4
MQMALLMNSSTSTHVLYPALKTEEPKTENMVSIFDSNLLQNQVNMPKEFIWPSMDLVNTAQEELK
EPLIDLSVMKSGDEEAIASAAELVRKACLKHGFFQVINHGVDQELINDAYCEVDPIFNLPINKKLSA
KRVHGGVSGYSGAHADRYSSKLPWKETFSFVYNHQNDSNSQIVNYFKSVLGEDFQQTGWVYQK
YCEAMKELSLVIMELLAISLGIDRLHYRRFFEDGDSIMRCNYYPPCKSSNLTLGTGPHSDPTSLTILH
QDQVGGLEVFADNKWVAVRPRPEALVINIGDTFMALSNGRYKSCLHRALVNRYRERRSLVFFVCPR
EDKVVRPPENLLSKNEARKYPDFTWSSLFEFTQKHYRADVATLQSFFQWHSSCNL
>MtGA20ox5
MDSGLCLVSSPNHQNIVQNNFYDPSWLQKQRNVPMNFVWPKEYLVNANEEFQAPLIDLDGFLKG
NEETTNNVAMLISKACSTHGFFQVINHGVDLSLIGEAYDQMDAFFKQPIDKKLIARKIKGSMWGYS
GAHADRFSSKLPWKETLSFPFHDNNVFEPSVTNYFDSTLGEDFQQTGVAFQKYCEAMKKLGMKL
MEILAISLGLDRFHFKSLFEDGCSIMRCNYYPSCQEPSVALGTGPHCDPTTLTILHQDQVGGLDVFA
DQKWQTVRPRSDAFVVNIGDTFTALSNGRYKSCLHRAVVNRYKERRSLAFFLCPKEDKMVRPSQD
IVSRDGTKQYPDFTWSQLLQFTQNHYRADEATLQNFTKWLLSSKITNTLP
>MtGA20ox6
MDSSQLLCELQIQSHVPKNFIWPKEYLEDAHEELQAPVVDLEGFLKGDNEATQHAAMLINEACLN
HGFFQVINHGVDLHLITQAYVQMDTIFKLPLHRKESVYKAPGSMWGYSGAHAHRFSSKLPWKETF
SFPYLENAVEPVVTNYFKSTLGDDFEQAGVTFEKYCNAMKELGMNLTELLAISLEVNDRLHYREL
FEEGCSIMRCNNYPCCKQPSLVLGTGPHCDPTSITLLHQDQVGGLQVFVDNKWHTVQPLPNAIVV
NIGDTFMALSNGRYKSCLHRAVVNQHKQRRSLAFFLCPKEDKVVRPPHDIISRDGTKQYPDFTWS
DLLEFTQNYYRADESTLSNFTNWLLSSKSQNFTLINK
>MtGA20ox7
MASNLKSENDFKGLIDFKSLHEEAKVPQEFIWSSEDLVETSKEELNVPVIDLEAIFNGDDAALAAA
AKIVRETCMEHGFFQVTNHGVDQNLIDATYQEFVSLFKLPLDRKLNAMRNPWGYSGAHAARYSA
SLPWKETFTFQYKHYDQSETQIVDFFTAALGDDHQHAGWVLQKYCEAMKKLTDVILELLAISLDV
DRSYYKKFFEDAETMMRCNSYPPCSGIHAGALGTGPHCDPTSVTILFQDQVGGLEAFVDNKWLGI
RPQPNNFVINIGDTFKALTNGVYKSCLHRVLANREKDRKTLAFFLCPKGDKIVRAPENILGRQEPTK
YPDFTWKQFFEFTQRKHRADPNTLPDFVSWINSNSSF
>MtGA20ox8
MESINTPSLPFFPPPKDQQGENKVQFFDFTLLQKEGNVPKEFIWPSEHWVKSSGENIELPLIDIGVIK
SDEAAMANAARIVREACIKHGAFEVTNIGVDSDFINAVLQETYNIFKLPLSKKITAIAKDSGFSVAH
AERYTTVLPWKETFTFMYKHNTKNETQVVDVVNSLLGEDFQQSGLVYQKYSDAMNDLTEVIMEL
LAISLGVDRKHYQRFFEDAESMMRCNFYPPCSANLTGALGNGPHCDPISITILLQDQVGGLEVFAD

NKWLAVPPKPDTFVINIGDTFMALTNGLYKSCLHRVLVSNELERKSLTFFLNPRGDKTVSPPNELLE
NEEARKYPDYKWSELYEYTQKTRRVDASTLDSFIAWRHSSETSKF
>MtGA3ox1
MPSLSEAYRAHPVHVNHKHPDFNSLQELPESYTWNHLDDHTLIKEGTTSSIVPVIDLNDPNASKLIG
HACKTWGVYQVVNHGIPISLLDEIQWLGQTLFTLPSHQKLKAIRSPDGVSGYGLARISSFFPKLMW
SEGFTIVGSPLDHFQQLWPQDYAKHCDTVLQYDEAMKKLAGKLMWLMLDSLGITMEDIKWAGSK
AQFDEKACAAMQLNSYPSCPDPDHAMGLAPHTDSTFLTILSQNDISGLQVQREGSGWVTVPPLHG
GLVVNVGDLFHILSNGLYTSVLHRVLVNRTRQRFSVAYLYGPPSNVEICPHEKLVGPTQPPLYRSVT
WNEYLGTKAKYFNKALSSVSLCAPINGLFDVNDSNKSSVQVG*
>MtGA3ox2
MATTLSEAYRDHPLHLHHIIPLDFSSFRTLPDSHAWPQSNDDGSDNFTSNGCYDNDDEDGSCIPIIDL
NDPNAMEQIGLACEKWGAFQLKNHGIPLNFIEEVEEEAKRLFSLPSKEKLKALRSAGGGTGYGRA
RISPFFPKFMWHEGFTIMGSPSNDAKKIWPNDYKRFCDTMENYQKQMKTLAEKLTNMILNILGISQ
EQNKWIGSNNHVGAMQLNFYPCCPDPKKAMGLAPHTDTSLFTILHQSQTNGLQLFKEGVGYVPV
DPHPNTLVVNTGDILHILSNSRFRCSLHRVVVNDISDRYSVAYFYGPPVDYLVSPCVGDNSLPRFRA
LTVKDYIGIKAKNLGGALSLISTLLDHDD*
>MtGA2ox1
MVVLSKTSLEQYPCIRNLKQTIFSTEIPMVDLSKPDAKNLIVKACEEFGFFKVINHGVSMKCISLLES
EAVKFFSMSIDQKEKAGPANPFGYGNKKIGQNGDIGWVEYLLLTNNQDFNQFKLSPAFGKDSDKL
RCLLSEYMSSVKKMGCEILELMASGLNIEENNVFSKLLMDKESDCIFRLNHYPPCPPKSNLNNNEN
ENVIGFGEHTDPQIISLLRSNNTSGLQIRLKDKSWISVPSDHNSFFVNVGDSLQVMTNGRFKSVRHR
VLANGFKSRLSMIYFGGPSLNEKIAPLPCLIKGNECLLYREFTWFEYKKSAYATRLSDNRLCHFERIK
DSS
>MtGA2ox2
MVLLSKPSSEQYTYVRNNMQATTFSSSIPLVDLSKPDAKSLIVKACEDFGFFKVINHGIPMEAISQLE
SEAFKFFSLPLTEKEKAGPANPFGYGNKRIGPNGDVGWVEYLLLNTNQEHNFSLHGKDIDKFRCLL
NDYKCAMRNMACEILDLMAEGLKIQPKNVFSKLVMDKQSDSAFRVNHYPACPELAINGENLIGFG
EHTDPQIISLLRSNNTSGFQISLRDGSWISVPPDHRSFFINVGDSLQVMTNGRFKSVRHRVLANGINP
RLSMIYFGGPPLSEKIAPLPSLMKGNESLYKEFTWFEYKNSTYGTRLADNRLGNYERIAAS
>MtGA2ox3
MVVLSKPTLNNFLLVKSCKPSTTLLNGIPVVDLADPEAKTLIVKACKEFGFFKVVNHGVPLEFMSN
LENEALRFFKKPQSEKDRAGPPDPFGYGSKRIGSNGDVGWVEYILLNTNPDVISNKSLSFYRENRQ
NLRSAVEDYIAAMKKMCCLVLELMADGLGIEPKNVLSRLLKDEKSDSCFRINHYPPCPEVQQAAL
NGRNLLGFGEHTDPQVISVLRSNSTSGLQICLTDGTWVSVPPDHTSFFINVGDTLQVLTNGRFKSVK
HRVLADTTKSRLSMIYFGGPPLSEKIVPLPSLMLKKEESLYKEFTWLEYKKAMYNSRLADYRLGPF
EKSYGK
>MtGA2ox4
MVVLSQQATLNELFHINTCKPTSYVFKGVPEVDLSDPEAKTLIVNACTEFGFFKVVNHQVPLELITN
LENETLKFFGQPQLEKEKAGPPDPFGYGSKIIGTNGDVGWVEYLLLNTNPDVISLKSLFLLQQNTK
NFRCAAEEYIVAVKEVCCEVLELMADGLRIEPRNVFSRLVRDERSDSCLRVNHYAACGELQALSGG
NLIGFGEHTDPQIISVLRSNNASGLQICLRDGTWVSIPPDHTSFFISVGDSLQVITNGELKSVKHRVLT
DTSMSRLSMIYFGGPPLNEKLVPLPSILGSKEQQSLYKEFTWREYKNAAYKSKLAYNRLSLFEK
>MtGA2ox5
MVVASPNSILGERIIPIDLPMIDLSAEKSMVIKLIVKACEEYGFFNVINHGVPHDIISKMEEVGFDFFA
KPMEQKKLVALGNPFGYGCKNIGFNGDMGEVEYLLLNANAPSIPNDSSNFSSSVSAYTEAVKELAC
EILELMAEGLGVPDTSIFSTFITQLDNDSLLRFNHYPPKDCKDRDNSNSYNVGFGEHSDPQILTILRS
NDVAGLQISLQHGVWNPVTPDPAAFCVNVGDLLEVMTNGRFVSVRHRAVTNSYKSRMSVAYFGA
PPLDACIVAPSVMVTPNRPSLLFKPFTWAEYKKVTYSLRLGDSRIDLFKNCTQIE
>MtGA2ox6
MVLASPKPMRNETILPNDLIPIVDLKSERSEVIKQIVKASEEYGFFKVINHGISDGTIEKMEEAGFSFF
AKPMSQKKQAAPAYGCKNIGFNGDIGEVEYLLLNANTSSIAQISKTISNDDPHSNFRCRVSEYTEAV
KEVACEILELMAEGLGVPDTKVFSSLIKDIDSDSVLRLNHYPPTLNKDKSHSNNVGFGEHSDPQILTI
LRSNDVSGLQISLQHGLWIPVNPDPEALCINIGDVLEVMTNGRFVSVRHRAMTNSYKSRMSMAYF
GAPPLNASIVAPPVLVTPTRPSLFRPFTWADYKKATYSLRLGDTRIQLFRANIA
>MtGA2ox7
MVVPSPTSMIRTKKTKAVGIPTIDLSMERSELSELVVKACEEYGFFKVVNHGIPKEVISRLENEGTEF

FSKNSTEKLQAGTSTPFGYGCKNIGPNGDKGDLEYLLLHTNPNSISERSKTIAKDHPIKFSCIVTDYI
EAVKELACEILELAAEGLWVPDKSSLSKVIKDVHSDSVLRINHYPPVKKLSKDNLDPSKFQNNNNTI
GFGEHSDPQILTILRSNNVGGLQISTQHGLWIPVHPDPNEFYVMVGDSLQVLTNGRFVSVRHRVLTN
TTKPRMSMMYFAAPPLNWWISPLSKMVTAHNPSLYKPFTWAQYKQAAYALRLGDSRLDQFKLQK
QEDNTHYHHDT
>MtGA2ox8
MDYEPPFLQTYMSLLQGTNDLISDLSDVEKREIPLIDLKRLKLDQLEREECMKEITEAARKWGFFQ
VVNHGVSQEVLKNMQFEEKEVFRTPFGIKSQENFLNLPSRTYRWGNASAINPKQLMWSEALHIFLP
DIEKMDQHKSLRSSIESFVKVVTPLAENLVQILAQELNINFSYFQQNCSANTSYLRLNRYPPCPFPSK
VIGLLPHADTSFITIVHQDHIGGLQLMKDGKWISVKPNSEALIVNVGDLFQALSNGLYTSVGHRVV
AAEKVERFSLAYFYGPSIDAVIESYATPPLYRKFTFGEYKEQTMKDLKEGGDKVGISRFLL
>MtGA2ox9
MDFEPPFLKIYNTLLEKNLGDNSENDLYSKVEGSEELPLIDLEKLNLEDPKREECMKEISEAASKWG
FFQIINHGISNEILNKMISEQKKLFYQPFVNKLSAETVFNLSPKTYRWGNPCATNLRQLSWSEAFHF
ALTDIPNMDQHITLRSSLEDFATRMDTLAENLVEILALKVNMKSNHFQENYLPKSSFIRLNRYPPCPI
SSEVFGLLAHCDTSFLTILYQDSVGGLQLMKDGKWVDVKPNPSALVVNIGDLFQALSNDVYKSIK
HRVVAAEEVERFSTAFFYCPFNDAVIQSENKPAVYKKFTLREYRQQTLNDVKETGDKVGLSRFVL
>MtGA2ox10
MGLIDSDPPFEETYKNLFNKDQNIINDELMVDNECELPVIDLSRLNDDDEVAREECKSMIANASQE
WGFLQVVNHGISSDILTRLRCEQKKVFKEPFDKKTKEDKFLNFSAGSYRWGTPTATCIKQLSWSEA
FHIPLTDILGSNTHLSSIIEQFATTVSNLAQILANILAEKLGHQSSFFKENCLPNTCYLRLNRYPPCPID
FRIHGLMPHTDSDFLTILYQDQVGGLQLVKDGKWVAVKPNPDALIINIGDLFQAWSNGVYKSVEHR
VVTNPRVERFSVAYFLCPSNDTMIESCKEPSIYRKFSFKEYRQQVRDDVQKLGSKIGLPRFIIN
>MtGA2ox11
MDPVSDPPFEEAYKILLNKTKNNRNNVPNDNKFPVVEECELPVIDLSRLEDDNEMVREACKYEIAR
ASQEWGFFQVINHGIPNDIFSRLKCEQEKVFKLPFDKKTKEDKFLQFSSGSYRWGTPSATCVGQLS
WSEAFHIPLKDVLESNAQPNTLRSTIEQFAIISSNLAQTLAHILAEKMGHESTYFKENCLPNTCYLRL
NRYPPCPIASEIHGLMPHTDSDFLTILYQDQVGGLQLVKDKKWIAVKPNPSALIINIGDLFQAWSNGL
YKSVEHRVVTNPKVERFSMAYFLCPSNESVIESCKKPSLYKEFSFQEYRQQVRDDVQKLGTKIGLP
RFLLF
>MtGA2ox12
MDPVSDPPFEEAYKILLNKTKNNRNNVPNDNKFPVVEECELPVIDLSRLEDDNEMVREACKYEIAR
ASQEWGFFQVINHGIPNDIFSRLKCEQEKVFKLPFDKKTKEDKFLQFSSGSYRWGTPSATCVGQLS
WSEAFHIPLKDVLESNAQPNTLRSTIEQFAIISSNLAQTLAHILAEKMGHESTYFKENCLPNTCYLRL
NRYPPCPIASEIHGLMPHTDSDFLTILYQDQVGGLQLVKDKKWIAVKPNPSALIINIGDLFQAWSNGL
YKSVEHRVVTNPKVERFSMAYFLCPSNESVIESCKKPSLYKEFSFQEYRQQVRDDVQKLGTKIGLP
RFLLF
>MtGA2ox13
MIDSNPPLLNHYGALLRNSAEPQKAKSSNGQDNTVVECELPLIDLNGLKSCNVSERLACTAAICKA
ASEWGFFQVINHGINPDLLRNMREEQMKLFRVPFEKKVTCGLLNNPYRWGTPSATSSNHFSWSEA
FHIPLTMISEAACWGEFNTLREAINEFAAAMLEVSRLLAGILAENLGHPTDAVEKLCDASTCFLRLN
HYPSCPKSKEEIFGLVPHTDSDFLTILYQDQVGGLQLMKDSKWVAVKPNPEALIVNIGDLFQAWSN
DEYKSVEHKVVANDKVERYSIAYFLCPSYTTMISGCKEPSTYKNFTFGEYRHQIQEDVKKIGHKVG
LSKFLRKDTYTTTMA
>MtGID1A
MAGSNQLNPNDSKMVVPLNMWVLISNFKLAYNLLRRPDGTFNRDLAEFLDRKVPANANPVDGVF
SFDVIVDRETNLLTRIYRPVEGEEQHVNIVDLEKPVTAEVLPVVMFFHGGSFAHSSANSAIYDTLCR
RLVGICNAVVVSVNYRRAPENRYPCAYEDGWKAVKWVNSRTWLQSKKDSKVHIYMVGDSSGGNI
VHHVALKALDSGIPVLGNILLNPLFGGEERTESEKRLDGRYFVRVKDRDWYWRAFLPEGEDRDHH
ACNPFGPKGRSLEGVAFPKSLVVVAGLDLVQDWQLGYAKGLEKAGQNVKLLFLEQATVGFYLLPN
NEHFSVVMDEIKHFVNSDCS
>MtGID1B
MRRHKRAKKTNIIDINMAGSNEVNLNESKSVVPLNTWVLISNFKLAYNLLRRADGTFNRELAEFL
DRKVPANTIPVDGVFSFDHVDRNSGLFNRVYQPAPENVTTWGIIELEKPLSTTEIVPVIIFFHGGSFSH
SSANSAIYDTFCRRLVSMCKAVVVSVNYRRSPEHRYPCAYEDGWNALNWVKSRTWLQSGKDSKV
YAYMAGDSSGGNIAHHVAVRAAEEDVEVLGNILLHPLFGGEKRTESEKKLDGKYFVRLQDRDWY

WRAFLPEGEDRDHPACNPFGPKGKSLVGLKFPKSLVCVAGLDLLQDWQLEYVEGLENSDQDVKLL
YLKEATIGFYFLPNNDHFYCLMNEINTFVHPNC
>MtGID1C
MTGTNEVNLSESRSVVPLNTYVLISNFKLAYNLLRRADGTFNRDLAEFLDRKVPANAIPVDGVFSF
DHIERNTGLFNRVYLPSSSENESQWGVKDLEKPLSTTEIVPVIVFFHGGSFSHSSANSAIYDTFCRRL
VSVCKAAVVSVNYRRSPEYRFPCAYEDGWNALKWVKSRKWLQSGKEKKVYVYMAGDSSGGNIV
HHVAVKACEEKAEGIEVLGNILLHPLFGGEKRTDSEMRLDGKYFVRLQDRDWYWRAFLPEGEDR
DHPACNPFGPKGEKNLKGLDKFPKSLVCVAGLDLLQDWQLAYVDGLRNFGQDVKLLYLKEATIGF
YFLPNNDHFYCLMEEIKNFVNPNC
>MtDELLA1
MWREEKETNGGGMDELLAALGYKVRSSDMADVAQKLEQLEMVMGSAQEEGINHLSSDTVHYD
PTDLYSWVQTMLTELNPDSSQINDPLASLGSSSEILNNTFNDDSEYDLSAIPGMAAYPPQEENTAA
KRMKTWSEPESEPAVVMSPPPAVENTRPVVLVDTQETGVRLVHTLMACAEAIQQKNLKLAEALV
KHISLLASLQTGAMRKVASYFAQALARRIYGNPEETIDSSFSEILHMHFYESSPYLKFAHFTANQAI
LEAFAGAGRVHVIDFGLKQGMQWPALMQALALRPGGPPTFRLTGIGPPQADNTDALQQVGWKL
AQLAQTIGVQFEFRGFVCNSIADLDPNMLEIRPGEAVAVNSVFELHTMLARPGSVEKVLNTVKKIN
PKIVTIVEQEANHNGPVFVDRFTEALHYYSSLFDSLEGSNSSSNNSNSNSTGLGSPSQDLLMSEIYL
GKQICNVVAYEGVDRVERHETLTQWRSRMGSAGFEPVHLGSNAFKQASTLLALFAGGDGYRVEE
NNGCLMLGWHTRSLIATSAWKLPQNESK*
>MtDELLA2
MKREHKLEHEDMSSGSGKSGVCWEDDGGGMDELLAVVGYKVKSSDMAEVAQKLEQLEQAMM
GNNFHDHDESTIAQHLSNDTVHYNPSDISNWLQTMLSNFDPQPNNPSVNSDDNDLNAIPGKAIYA
ADEFTSRKRVKRNESVTVTTESTTTRPIMVVETQEKGIRLVHSLMACAEAVEQNNLKMAEALVKQ
IGYLAVSQEGAMRKVATYFAEGLARRIYGVFPQHSVSDSLQIHFYETCPNLKFAHFTANQAILEAF
QGKSSVHVIDFSINQGMQWPALMQALALRPGGPPAFRLTGIGPPASDNSDHLQQVGWRLAQFAQT
IHVQFEYRGFVANSLADLDASMLELRSPETESVAVNSVFELHKLNARPGALEKVFSVIRQIRPEIVT
VVEQEANHNGPAFLDRFTESLHYYSTLFDSLEGSSVEPQDKAMSEVYLGKQICNVVACEGTDRVE
RHETLNQWRNRFNSAGFSPVHLGSNAFKQASMLLALFAGGDGYKVEENDGCLMLGWHTRPLIAT
SAWKLAAANSVVVSH*
>MtDELLA3
MEIVSDSSSPSKTKDIDGLLANVGYKVRSSELHQVAQNLERLESAIVNSSSDISQFASDTVHYDPSDI
GNWVDNLLSEFDHTASLPYDFSQLPDLSAPPPQHHHHQSSSTVNNVEEDSAIKLVHMLMTCADSV
QRGNLSLAGSLIEGMQGLLANMNTNSGIGKVAGYFIDALNRRIFGQNNVSHQVSLYENDVLYHHY
YEACPYLKFAHFTANQAILEAFNGHDCVHVIDFNLMHGLQWPALIQALALRPGGPPFLRLTGIGPPS
PDDRDNLREIGLRLAELARSVNVRFAFRGVAAWRLEDVKPWMLQVSSKEAVAVNSIFQLHRLLGSE
SDSNYHSGIEMVLGWIRSLNPKIVTVVEQEANHNENGFMERFTEALHYYSTVFDSLEACPVEPDK
AMAEMYLQREICNVVCCEGPARVERHEPLVKWKERLGKAGFRPLHLGSNAFKQASMLLTLFSAE
GYCVEENQGSLTLGWHSRPLIAASAWQAVPLLDAETLRFDC*
